# High Rate of Simian Immunodeficiency Virus (SIV) Infections in Wild Chimpanzees in Northeastern Gabon

**DOI:** 10.3390/v7092855

**Published:** 2015-09-15

**Authors:** Vanina Boué, Sabrina Locatelli, Floriane Boucher, Ahidjo Ayouba, Christelle Butel, Amandine Esteban, Alain-Prince Okouga, Alphonse Ndoungouet, Peggy Motsch, Guillaume Le Flohic, Paul Ngari, Franck Prugnolle, Benjamin Ollomo, François Rouet, Florian Liégeois

**Affiliations:** 1 UMI 233 “TransVIHMI”, IRD / UM-INSERM U1175/ UM1, 34394 Montpellier, France; svetnina@yahoo.fr (V.B.); sabrina.locatelli@ird.fr (S.L.); floriane.boucher@wanadoo.fr (F.B.); ahidjo.ayouba@ird.fr (A.A.); christelle.butel@ird.fr (C.B.); amandine.esteban@ird.fr (A.E.); 2 Centre International de Recherches Médicales, BP 769 Franceville, Gabon; aokouga@yahoo.com (A.-P.O.); alphonsendoungouet@gmail.com (A.N.); p.motsch@yahoo.fr (P.M.); leflohg@yahoo.fr (G.L.F.); paul_ngari@yahoo.fr (P.N.); franck.prugnolle@ird.fr (F.P.); bollomo@yahoo.fr (B.O.); franrouet@yahoo.fr (F.R.); 3 Laboratoire Maladies Infectieuses et Vecteurs: Ecologie, Génétique, Evolution, Contrôle, UMR 224 IRD/CNRS/UM1, 34394 Montpellier, France; 4 Institut Pasteur du Cambodge, Phnom-Penh BP 983, Royaume du Cambodge

**Keywords:** Chimpanzee, SIV, AIDS, Africa, antibody, microsatellite, primate, prevalence, retrovirus, transmission

## Abstract

The emergence of HIV-1 groups M, N, O, and P is the result of four independent cross-species transmissions between chimpanzees (cpz) and gorillas (gor) from central/south Cameroon and humans respectively. Although the first two SIVcpz were identified in wild-born captive chimpanzees in Gabon in 1989, no study has been conducted so far in wild chimpanzees in Gabon. To document the SIVcpz infection rate, genetic diversity, and routes of virus transmission, we analyzed 1458 faecal samples collected in 16 different locations across the country, and we conducted follow-up missions in two of them. We found 380 SIV antibody positive samples in 6 different locations in the north and northeast. We determined the number of individuals collected by microsatellite analysis and obtained an adjusted SIV prevalence of 39.45%. We performed parental analysis to investigate viral spread between and within communities and found that SIVs were epidemiologically linked and were transmitted by both horizontal and vertical routes. We amplified *pol* and *gp41* fragments and obtained 57 new SIVcpz*Ptt* strains from three sites. All strains, but one, clustered together within a specific phylogeographic clade. Given that these SIV positive samples have been collected nearby villages and that humans continue to encroach in ape’s territories, the emergence of a new HIV in this area needs to be considered.

## 1. Introduction

The four groups of Human Immunodeficiency Virus Type 1 known to date (HIV-1 M, N, O and P) are the result of four independent cross-species transmission events between apes and humans in west-central Africa [1,2,3,4,5]. During the last decade, the development of serological techniques and the improvement of viral RNA amplification in faecal samples provided the possibility to perform large-scale studies on wild great apes across equatorial Africa, as previously demonstrated in Cameroon, Democratic Republic of Congo (DRC) and Tanzania [4,5,6,7]. These studies brought to light the origins of HIV-1 group M and N in chimpanzees (*Pan troglodytes troglodytes* (*Ptt*)), infected with SIVcpz*Ptt* [1] and HIV-1 group O and P in gorillas (*Gorilla gorilla gorilla*), infected with SIVgor [2,3] from Cameroon. The long-term monitoring of habituated chimpanzee communities from east Africa (*Pan troglodytes schweinfurthii* (*Pts*)) revealed that chimpanzees infected with SIVcpz*Pts* could develop an AIDS-like pathology [8]. SIV infects more than 45 species of Non-Human Primates (NHP) living in Africa [9,10] but the view that SIV is apathogenic in all of their natural hosts [11] has been challenged by studies showing that chimpanzees naturally infected with SIV do develop an AIDS-like syndrome [12,13,14]. These findings provided compelling evidence that SIVcpz has a substantial negative impact on the health, reproduction and lifespan of chimpanzees in the wild [8,12].

The SIVcpz*Ptt* ancestors of HIV-1 group M and N have been isolated from chimpanzees inhabiting southeastern Cameroon (close to the Central African Republic (CAR) border) and south-central Cameroon (inside the Dja reserve), respectively [1]. The closest relatives of HIV-1 group O and P are SIVgor strains from wild-living gorillas inhabiting central and southwest Cameroon, respectively [2,3]. Group M, the HIV-1 pandemic form, has so far infected at least 60 million people and caused more than 25 million deaths [15], whereas groups N and P are found exclusively in Cameroon with only a few infected individuals [16,17]. HIV-1 group O is mainly present in west Central Africa with low prevalence rates (<0.5% of HIV infected individuals) [18,19,20].

There are four chimpanzee subspecies [21]. However, only two of them—*Pan troglodytes troglodytes,* ranging from Cameroon, south of the Sanaga River, to the Congo River/Ubangi River (DRC) and *Pan troglodytes schweinfurthii*, ranging from the Ubangi River/Congo River in CAR and DRC, to western Uganda, Rwanda and western Tanzania - are infected with SIVcpz*Ptt* and SIVcpz*Pts,* respectively [22]. SIVcpz infections are absent in the other two chimpanzee subspecies [5,23,24], *Pan troglodytes verus* from West Africa and *Pan troglodytes ellioti* inhabiting Nigeria and western Cameroon, north of the Sanaga River [25]. Past studies showed that SIVcpz infecting neighboring populations is phylogenetically interspersed, whereas chimpanzee populations that are separated by major geographical barriers (such as rivers) or by long distances are infected with SIVcpz belonging to distinct lineages [1,5].

SIVcpz infection rate is unevenly distributed, ranging from 30% to 50% in certain communities, whereas other communities are uninfected [1,5,26]. It must be pointed out that sampling did not cover all regions in Africa where chimpanzees live and many areas remain today totally unexplored. For instance, no study on SIV in wild chimpanzees has been conducted in Gabon (Gab) despite the fact that the first characterized SIVcpz*Ptt* strains were isolated in the north and eastern part of this country at the end of the 1980s. They were named SIVcpzGab-1 and Gab-2 [27,28]. More recently, a third SIVcpz strain, SIVcpzGab-4, was described in a wild-born chimpanzee held captive at the Centre International de Recherches Médicales de Franceville (CIRMF) [29]. In addition, information about SIV infections in other sympatric NHP populations is also scarce: a recent study highlighted the presence of SIV infection in red capped mangabeys (*Cercocebus torquatus*), mandrills (*Mandrillus sphinx*) and moustached monkeys (*Cercopithecus cephus*) bushmeat collected at local markets in Gabon [30,31]. Before the above-mentioned study, a semi-free ranging population of mandrills has been studied in Gabon for SIV infection and its patterns of transmission [32,33].

The aims of this study where therefore to (1) conduct the first large-scale survey in Gabon to further document the prevalence and genetic diversity of the SIV virus infecting wild chimpanzee populations, and (2) determine the routes of viral transmission and estimate their spread across territories by investigating the parental links among and between groups.

## 2. Materials and Methods

### 2.1. Study Sites and Chimpanzee Faecal Samples Collection

**Figure 1:**
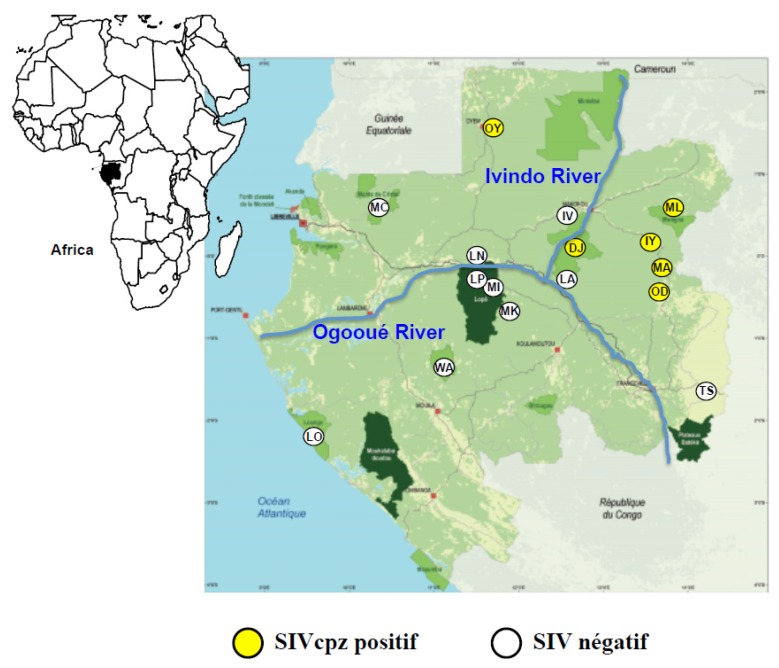
Geographical distribution of chimpanzee faecal samples collection sites (white and yellow circles) from Gabon Locations of faecal samples collection: LO = Louango, WA = Waka, MK = Makande, MI = Mikongo, LP = Lopé National Park, LN = Lopé SEGC site, MC = Monts de Cristal, OY = Oyem, IV = Ivindo, DJ = Djidji, LA = Langoué, TS = Tsouba, OD = Odjala, Ma = Makatamangoye, IY = Iyoko milieu, ML = Malouma. Yellow circles represent sites where SIVcpz positive samples were identified.

Between September 2009 and June 2013, we collected faecal samples from wild chimpanzees living in remote primary forests and in secondary forests surrounding villages in Gabon, north and south of the Ogooué River (Figure 1). Northeast of the river, we collected stool specimens outside of villages built along the trail connecting Odjala village (OD) to Malouma village (ML). The distance between OD and Makatamangoye 2 (MA) and MA and Iyoko milieu (IY) is about 30 km, walking-distance, whereas IY and ML are located approximately 60 km apart (straight line) (Figure 1).

We collected faecal samples underneath night nests or on track. We recorded GPS positions and estimated the time of faecal deposition, by assessing the texture, stage of decomposition and presence of flies on the dung. We inferred the species origin in the field according to shape, size and texture of the faecal samples as well as presence of footprints or nests nearby. We collected about 20 g of dung in a 50 mL tube, containing 20 mL of RNA*later*® (Ambion, Austin, TX, USA), and we kept samples at ambient temperature at base camp, for a maximum of three weeks. Samples were then stored at −80°C in Franceville, Gabon.

All NHP samples were obtained with the authorization of the Provincial Inspections of Water and Forests and the Centre National de la Recherche Scientifique et Technologique (CENAREST, AR0031/09, AR0006/11). 

### 2.2. Detection of SIVcpz Antibodies in Chimpanzee Faecal Samples

All faecal samples were tested for the presence of HIV-1 cross-reactive antibodies using the INNO-LIA^®^ HIV I/II score Confirmation test (Fujirebio, Courtaboeuf, France), as previously described [34]. This test contains HIV-1 and HIV-2 recombinant proteins and synthetic peptides coated as discrete lines on a nylon strip. These antigens can cross-react with SIV antibodies present in the sample. Faecal samples stored in RNA*later*^®^ must undergo dialyses before IgG antibodies can be recovered. We therefore applied dialysis methods previously adopted for antibody detection in fecal samples from gorillas and chimpanzees [1,2]. We performed all assays according to the manufacturer’s instructions. Samples were scored as INNO-LIA positive when they reacted with ≥1 HIV antigen.

### 2.3. DNA and RNA Extraction from Chimpanzee Faecal Samples

We used a QIAamp Stool DNA Mini kit (Qiagen, Valencia, CA, USA) to extract fecal DNA for species confirmation and microsatellite analyses, as published elsewhere [1]. Briefly, 2.0 mL of fecal RNA*later*^®^-preserved sample were re-suspended in stool lysis buffer, clarified by centrifugation, treated with an InhibitEx tablet, and proteinase K, and passed through a DNA binding column. Bound DNA was eluted in 100–200 μL of elution buffer.

Faecal RNA was extracted using the NucliSens Magnetic Extraction kit (Biomérieux, Craponne, France), which utilizes magnetic silica particles to purify RNA [35] following the same protocol steps adopted in previous studies [7]. Briefly, 2.0 mL of sample were vortexed with 7.0 mL of NucliSens Lysis buffer for 1 min. Samples were then incubated at room temperature for 1–2 h and centrifuged at 4000 rpm for 30 min. Supernatant were filtered and centrifuged at 4500 rpm for 5 min. We then followed the magnetic extraction procedure according to the manufacturer instructions to obtain a final elution volume of 50 μL fecal RNA.

### 2.4. Species Determinations

The species was confirmed by mtDNA analyses, as described previously [1,2,36]. Briefly, 5 µL of extracted faecal DNA were used for mitochondrial DNA amplification. A 450 to 500bp mtDNA fragment spanning the hypervariable D loop was amplified from faecal DNA using primers L15997 and H16498 [37]. Differences in DNA amplicon weight allowed us to determine whether faecal samples were collected from gorillas (450bp) or chimpanzees (500bp). When the d-loop amplification failed, a 386bp fragment spanning the 12SrRNA gene was targeted, using primers 12S-L01091 and 12S-H01478 [37]. If both amplification strategies yielded no results, samples were considered degraded. The samples origin was assessed with the “*Basic Local Alignment Search Tools*” [38].

### 2.5. Number of Collected Individuals

In order to determine the number of collected individuals, we ran microsatellite analyses on all SIVcpz positive and negative faecal samples from OD, MA and IY and on a randomly selected number of samples from ML (*n* = 90) (Table 1). Samples were genotyped at seven autosomal microsatellite loci with two multiplex PCRs, according to the protocols used in previous studies [4,7]. For sex determination, a region in the amelogenin gene that contains a deletion in the X, but not in the Y chromosome was amplified [39]. We used the Taq DNA polymerase Core kit (MP Biomedical, Irvine, CA, USA) with 2 to 10 μL fecal DNA. Homozygous loci were amplified from three to seven times to minimize problems associated with allelic dropout, which frequently occurs when genotyping low-yield DNA samples [40]. All PCR reactions included negative control samples for quality assurance. We analyzed each multiplex PCR product on an ABI 3130 capillary array genetic analyzer (Applied Biosystems, Foster City, CA, USA). We determined fragment sizes against a Genescan 600 Liz size standard (Applied Biosystems, Foster City, CA, USA), and allele sizes using the Genemapper ID version 4 software (Applied Biosystems, Foster City, CA, USA). We discarded all samples with unsuccessful amplification after five PCR attempts and two independent DNA extractions. We discarded from further analyses all samples that displayed an incomplete allelic profile (less than four loci), a multiple peak profile for the same locus, or discordant results.

**Table 1:** Crude and corrected estimations of Simian Immunodeficiency Virus (SIV) infection rate in wild chimpanzees from different field sites in Gabon (2009–2013).

					HIV Antibody Positive Profile				HIV Antibody Negative Profile				
Collection Sites	Faecal Samples Collected	SIVcpz Antibody Positive Samples	Estimated Number Of Individuals Collected	Samples Successfully Genotyped/not Genotyped	Prevalence Of SIVcpz Antibody Positive (%) Genotyped Animals (Individuals)	vRNA Positive Genotyped Animals *pol* / *env^A^*	SIVcpz Antibody Positive Samples	Number Of vRNA Positive Samples *pol* / *env^B^*	Antibody Negative Samples Tested By PCR	Number Of Individuals Tested By PCR	Number Of vRNA Positive Individuals *pol* / *env^C^*	Samples Tested By PCR	Number Of vRNA Positive Samples *pol* / *env^D^*
LP	54	0											
MI	194	0											
TS	13	0											
MK	11	0											
LA	16	0											
MA	608	263 (44%)	224	504/104	103 (46%)	23/18	44	6/4	146	50	5/6	23	2/1
MC	33	0											
IV	19	0											
DJ	40	8 (20%)											
ML	181	85 (47%)	19	85/96	12 (63.5%)	5/1	47	5/2					
WA	18	0											
LN	12	0											
OY	78	1 (1.3%)											
IY	31	18 (58%)	12	25/6	4 (33.3%)	1 / 0							
OD	39	5 (12.8%)	17	35/4	4 (23.5%)	0							
LO	111	0											
Total	1458	380 (26,1)	272	649/210	123 (41.6%)	29/19	91	11/6	146	50	5/6	23	2/1

**^A^** In MA and ML sites 11 and 1 SIV strains were both amplified in *pol* and *env* respectively. **^B^** In MA and ML site 2 and 2 SIV strains were both amplified in *pol* and *env* respectively. **^C^** 5 SIV strains were both amplified in *pol* and *env*. **^D^** 1 strains were both amplified in *pol* and *env.*

We used Cervus v3.0 (Field Genetics, London, UK) [41] to assess allele frequency, expected heterozygosity, polymorphic information content (PIC), Hardy-Weinberg (HW) equilibrium, to identify samples collected in MA, ML, IY and OD that have matching genotypes, and to determine the suitability of loci to run downstream analysis including parentage analysis. We checked all genotypes mismatching at one locus for allelic dropout [42] and we allowed an allelic mismatch at one locus, but only if it represented a missing allele. We gave a consensus ID to matching samples (Table S1 supplementary material). We calculated P(ID)HW for each individual (Data not shown). P(ID)HW is the probability that two individuals drawn at random from a population will have the same genotype at multiple loci, assuming that the loci we chose for these analyses are inherited independently (*i.e.,* that they are unlinked). For parentage analysis, we assumed that we collected and successfully genotyped a quarter of the offspring in every group and a quarter of the candidate parents. The frequency of typing errors and the error rate in likelihood calculations were set at 0.01. The minimum number of typed loci was set at four out of seven. Allele frequencies, critical to run simulations and to assess confidence of parentage assignment were also calculated. Confidence in assignment of parentage to the most likely candidate parent is evaluated relative to a critical threshold determined in the simulation (LOD score). To ensure satisfactory confidence in individual parentage assignments, we regarded 95% as a minimum value for the population confidence level set in Cervus.

### 2.6. SIVcpz Partial pol, env and gp41-nef PCR Amplifications

RT-PCR amplifications were done for all SIV antibody positive samples, as well as for some SIV negative antibody samples from the MA site. We first amplified partial *pol* (400 bp) and *gp41* (450 bp) PCR fragments using consensus primers previously described (Table S2 supplementary material) [1]. In order to optimize our PCR amplification systems, we designed from the newly obtained *pol* and *gp41* sequences more specific primers allowing the amplification of 150 bp and 170 bp in *pol* and *gp41* respectively (Table S2). We then proceeded to amplify a larger PCR fragment (916 bp) spanning the end of gp41 and the first part of *nef* gene using a combination of specific and consensus primers (Table S2). In addition, we designed new consensus primers, which were used in combination with the specific primers to amplify a 365 and 325 bp nucleic acid fragments in *pol* and *env*, respectively (Table S2). For the first PCR round, bovine serum albumin (BSA) was added at the final concentration of 0.2 µg/mL to improve the success of amplification. All RT-PCR reactions were performed using the Expand Reverse transcriptase, and the Expand long Template PCR kit (Roche Diagnostics, Indianapolis, IN, USA), according to the manufacturer’s instructions. After purification with the Geneclean Turbo Kit (Qbiogene, Inc., Carlsbad, CA, USA), PCR products were sequenced on an automated sequencer (3130xl Genetic Analyser, Applied Biosystems, Foster City, CA, USA). The resulting sequences were aligned with SEQMAN DNASTAR software (Lasergene, DNASTAR, Inc., Madison, WI, USA).

### 2.7. Phylogenetic Analyses

The partial nucleic acid sequences of the new SIVcpz strains were aligned using MEGA 5 [43] with minor manual adjustments. We excluded those sites that could not be unambiguously aligned. We inferred the phylogenies for the small *pol* and *env* fragments using the Neighbor-Joining method (implemented in Mega 5 with the K2P model of evolution), and the Maximum Likelihood (ML) method (implemented in PhyML) for the 916 bp *gp41-nef* fragment and the 365 and 325 bp nucleic acid fragment in *pol* and *env,* respectively [44]. We tested the reliability of branching orders using the bootstrap approach (1000 replicates). The suited evolution model (GTR+Γ_4_+I) used in the ML analyses was defined using Topali [45].

### 2.8. Nucleotide Sequence Accession Numbers

The new sequences have been deposited to the GenBank under the following accession numbers: KR150263 to KR150368.

## 3. Results

### 3.1. SIV Infection in Wild Chimpanzees in Gabon

#### 3.1.1. Crude SIV Prevalence Rates in Wild Chimpanzee Faecal Samples

Between 2009 and 2013, we collected 1458 faecal samples from wild chimpanzees in 16 different locations across Gabon (Figure 1). Five locations were south and 11 north of the Ogooué River (Figure 1). We confirmed by mtDNA analysis that all faecal samples collected were from chimpanzees. Among the 1458 chimpanzee faecal samples collected in six different locations in the north and northeast of the country, 380 samples were SIV antibody positive, yielding an overall prevalence rate of 26.1% (0%–58%) (Table 1).

**Figure 2:**
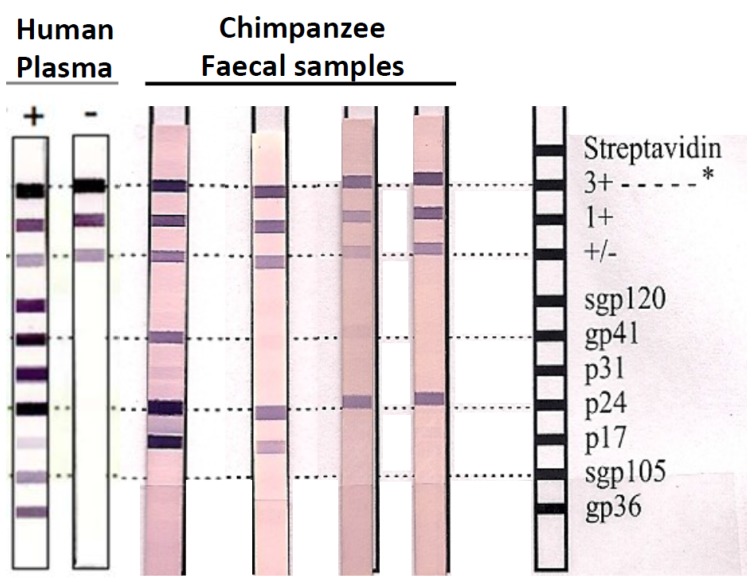
SIV antibody positive profiles. Example of HIV-1/HIV-2 cross-reactive antibodies profile in chimpanzee faecal samples using a line immunoassay (INNO-LIA HIV Confirmation, Innogenetics, Ghent, Belgium). Varying patterns of reactivity to HIV peptides and proteins (gp41, p24, p31 and p17) are shown. Plasma samples from HIV-1/HIV-2-negative and -positive persons are shown as controls on the left. The 3+, 1+ and +/− bands at the top of all test strips control for sample addition (presence of plasma immunoglobulin) and test performance (binding of secondary antibody).

The SIVcpz antibody positive samples were unevenly distributed with prevalence rate oscillating between 1.3 % and 58% according to the collection site (Table 1). In OY (north Gabon), only one specimen (on a total of 78 samples, for an infection rate of 1.3%) cross-reacted weakly with the HIV p24 antigen (Table 1, Figure 1). In DJ (Ivindo National Park), eight of 40 samples (20%) cross-reacted with HIV antigens (Table 1, Figure. 1). The highest sero-prevalence rate was obtained in three sites located in the northeast: ML, IY and MA, with respectively 47%, 58% and 44% of SIV infections (Table 1, Figure. 1). In addition, 12.8 % of the samples collected in OD, which is located 30 km south of MA, tested positive for SIV antibodies and presented antibody profiles comparable to those observed in DJ, ML, IY and MA (Figure 2). The Inno-LIA patterns were HIV p24 alone or p24 + p17 or, p24 + p17 + gp41 (Figure 2).

#### 3.1.2. Adjusted SIV Prevalence Rates According to the Number of Collected Individuals

**Figure 3:**
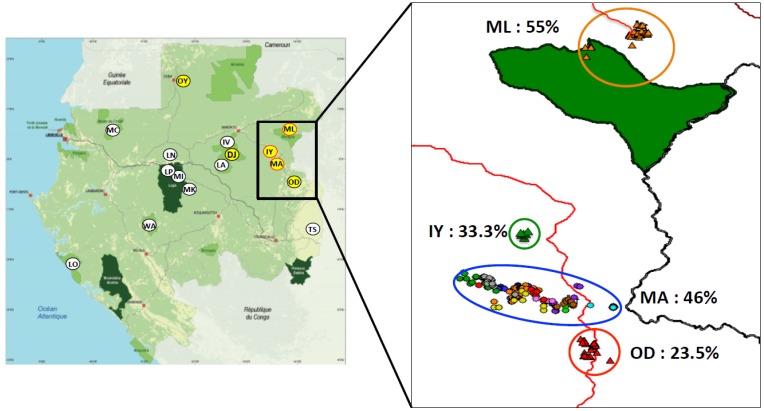
SIVcpz antibody positive samples from north-east Gabon. A/ Yellow circles with red perimeter represent sampling sites where SIVcpz have been PCR amplified. B/ details of SIVcpz positive sampling sites: colored circles correspond to the color codes used to represent the different SIVcpz identified in Figure 4 and Figure 5: Blue = MA, Green = IY and Orange = ML. Triangles correspond to SIVcpz antibody positive faecal samples collected in OD, IY and ML. Dots correspond to SIVcpz antibody positive faecal samples collected in MA. Prevalence based on serological tests is showed for each site. The trail linking Odjala to Malouma is represented by a red line. The green area corresponds to the different national parks. The site of DJ is not represented.

To study more in detail the SIVcpz prevalence in chimpanzee communities from the northeast, 13 follow-up missions were conducted in MA and three in ML (Figure. 3). From the 608 samples collected in MA, 504 samples were successfully genotyped, and corresponded to 224 individuals (Table 1). Microsatellite results were non-interpretable in the 104 remaining specimens. Thus, each chimpanzee was sampled 2.2 times. Among these 224 individuals, 103 were SIV antibody positive, leading to an infection rate of 46% (Figure 3, Table 1). Also, 39 of them were collected repeatedly (one to four times) during the various missions, within a 25 months period of time. Interestingly, the serological status of 10 out of 39 individuals was discordant (Table 2). For five animals (Mac-ID-010, -054, -055, -056, -058, -077) the first sample tested negative, according to serological tests, whereas the subsequent samples tested positive. An initial positive serological result followed by a negative result was obtained in four animals. In addition, samples from the same individual (Mac-ID-025, -044, -071, -150, -171) collected during the same field mission showed discordant serological results over time (Table 2). In ML, on 181 collected faecal samples (Table 1), 85 were successfully genotyped allowing 19 individuals to be identified. Each chimpanzee was thus sampled 4.5 times. Out of the 19 individuals, 12 were SIV antibody positive (63.5%) (Figure 3, Table 1). In IY, on 25 collected and genotyped samples (Table 1), 12 individuals were identified, meaning that each animal was sampled 2.1 times. Four out of 12 individuals were SIV antibody positive (33.3 %) (Figure 3, Table 1). In OD, 5 out of 39 faecal samples collected were SIV antibody positive (12.8%) (Table 1). We genotyped them all and we obtained 17 individuals, with each animal sampled 2.3 times. Four out of 17 individuals were SIV antibodypositive (23.5 %) (Figure 3, Table 1).

Altogether, these results lead to a high SIV prevalence in wild chimpanzee communities from eastern Gabon, with a mean corrected infection rate of 39.45% (23.5%–55%).

**Table 2:** Discordant serological test results in samples from the same individual.

	ID^a^	Field Number	Date of Sampling	SIV Serological Test^b^	GPS Points	
**Samples collected during different field missions**	**MA-ID-010 **	Gab-0902	07/04/11	neg	S00°08.276	E013°38.105
		Gab-1001	17/05/11	pos	8 Km to the west from MA village	
	**MA-ID-028 **	Gab-1012	20/05/11	pos	8 Km to the west from MA village	
		Gab-2092	08/07/12	neg	S00°08.170	E013°37.466
	**MA-ID-036**	Gab-1227	04/11/11	pos	S00°07.707	E013°31.226
		Gab-1228	04/11/11	pos	S00°07.707	E013°31.226
		Gab-1229	04/11/11	pos	S00°07.707	E013°31.226
		Gab-1230	04/11/11	pos	S00°07.707	E013°31.226
		Gab-1510	11/03/12	pos	S00°06.500	E013°31.722
		Gab-1513	11/03/12	pos	S00°06.397	E013°31.777
		Gab-1515	11/03/12	neg	S00°06.397	E013°31.777
		Gab-1517	11/03/12	pos	S00°06.335	E013°31. 788
		Gab-1518	11/03/12	pos	S00°06.264	E013°31.792
	**MA-ID-054**	Gab-0923	07/04/11	neg	S00°08.093	E013°36.047
		Gab-0924	07/04/11	neg	S00°08.093	E013°36.047
		Gab-1283	01/12/11	pos	S00°07.800	E013°35.962
		Gab-1288	01/12/11	pos	S00°07.890	E013°36.116
		Gab-1289	01/12/11	pos	S00°07.891	E013°36.128
		Gab-1295	01/12/11	pos	S00°08.002	E013.36.262
		Gab-1321	03/12/11	pos	S00°08.832	E013°35.001
	**MA-ID-055**	Gab-0926	07/04/11	neg	S00°08.193	E013°34.907
		Gab-1293	01/12/11	pos	S00°07.992	E013°36.273
		Gab-1294	01/12/11	pos	S00°07.986	E013°36.264
	**MA-ID-056**	Gab-0922	07/04/11	neg	S00°08.093	E013°36.047
		Gab-1297	01/12/11	pos	S00°08.086	E013°36.337
	**MA-ID-058**	Gab-0915	13/04/11	neg	S00°07.276	E013°35.538
		Gab-0929	08/04/11	neg	S00°08.552	E013°34.845
		Gab-1314	02/12/11	pos	S00°08.821	E013°36.022
		Gab-2488	06/05/13	pos	S00°08.790	E013°38.036
		Gab-2489	06/05/13	pos	S00°08.790	E013°38.036
	**MA-ID-077**	Gab-0919	07/04/11	neg	S00°08.046	E013°36.073
		Gab-1301	02/12/11	pos	S00°07.910	E013°36.707
**Samples collected during the same field missions**	**MA-ID-081**	Gab-0905	08/04/11	pos	S00°08.276	E013°38.105
		Gab-0989	04/05/11	neg	8 Km to the west from MA village	
		Gab-1010	20/05/11	neg	8 Km to the west from MA village	
		Gab-1011	20/05/11	neg	8 Km to the west from MA village	
		Gab-1016	20/05/11	pos	8 Km to the west from MA village	
		Gab-1018	20/05/11	neg	8 Km to the west from MA village	
		Gab-2486	06/05/13	neg	S00°08.790	E013°38.036
	**MA-ID-199**	Gab-2294	19/02/13	pos	S00°11.355	E013°48.664
		Gab-2305	04/03/13	neg	S00°11.352	E013°40.754
		Gab-2334	04/03/13	pos	S00°11.239	E013°40.902
	**MA-ID-025**	Gab-0977	02/05/11	pos	3 Km to the southwest from MA village	
		Gab-0978	02/05/11	pos	3 Km to the southwest from MA village	
		Gab-0981	02/05/11	neg	3 Km to the southwest from MA village	
	**MA-ID-044**	Gab-1275	01/12/11	pos	S00°07.720	E013°35.734
		Gab-1303	02/12/11	neg	S00°08.021	E013°35.394
	**MA-ID-071**	Gab-1350	03/12/11	pos	S00°10.331	E013°34.642
		Gab-1357	03/12/11	neg	S00°10.323	E013°34.658
		Gab-1358	03/12/11	neg	S00°10.328	E013°34.660
		Gab-1359	03/12/11	neg	S00°10.329	E013°34.653
		Gab-1360	03/12/11	neg	S00°10.334	E013°34.656
		Gab-1361	03/12/11	neg	S00°10.326	E013°34.658
		Gab-1362	03/12/11	neg	S00°10.330	E013°34.656
		Gab-1363	03/12/11	neg	S00°10.327	E013°34.656
		Gab-1364	03/12/11	neg	S00°09.555	E013°34.728
	**MA-ID-150**	Gab-2522	09/05/13	pos	S00°07.265	E013°30.113
		Gab-2523	09/05/13	neg	S00°07.265	E013°30.113
	**MA-ID-171**	Gab-2499	07/05/13	neg	S00°08.040	E013°37.291
		Gab-2505	07/05/13	pos	S00°08.040	E013°37.291
		Gab-2506	07/05/13	pos	S00°08.040	E013°37.291
		Gab-2511	08/05/13	pos	S00°08.040	E013°37.291
		Gab-2515	08/05/13	pos	S00°08.040	E013°37.291
		Gab-2517	08/05/13	pos	S00°08.040	E013°37.291
		Gab-2519	08/05/13	neg	S00°08.040	E013°37.291

^a^ Samples underligned have been collected during the same field mission; ^b^ neg = negative sample, pos = positive sample

### 3.2. Partial SIV pol, env and gp41-nef PCR Amplifications and Phylogenetic Analyses

In order to confirm whether the animals with HIV-like antibodies were infected with SIV, we ran PCRs using generic and specific primers in the *pol* (150bp) an*d gp41* (170bp) regions of the genome. Among the 123 individuals, which displayed a HIV-like antibody positive profile, and which were collected at three different sites (MA, ML, and IY), we amplified partial *pol* and/or *gp41* fragments in 29, five and one individual, respectively (Table 1). We also amplified *pol* and/or *gp41* fragments in eight samples from MA and in five from ML, which did not display a complete microsatellite profile (Table 1). All PCR attempts performed on samples from DJ, OD and OY were unsuccessful.

In addition, we ran these PCRs on a subset of 146 faecal samples from MA displaying a HIV-like negative profile. Of 146 samples, we identified 50 individuals by microsatellite analysis and we amplified seven *pol* and/or *gp41* fragments, which represented 14 % of infection rate with SIVcpz, whereas 23 out of 146 samples were non-interpretable by microsatellite analysis. However, we were able to amplify two SIVcpz fragments for two of them (Table 1). Altogether, using both *pol* and *gp41* PCR protocols, we amplified 57 new SIVcpz*Ptt* strains: 42 from genotyped chimpanzees and 15 from non-genotyped samples (Table 1). We also amplified a 916 bp fragment covering the gp41 and the 5′ half part of *nef* gene, as well as a 365 *pol* and a 325 bp *env* fragment for 14 SIVcpz strains from MA and/or ML. Only five SIVcpz strains were amplified with the three PCR systems, whereas three strains were obtained with the *pol* and *env* PCR systems.

**Figure 4:**
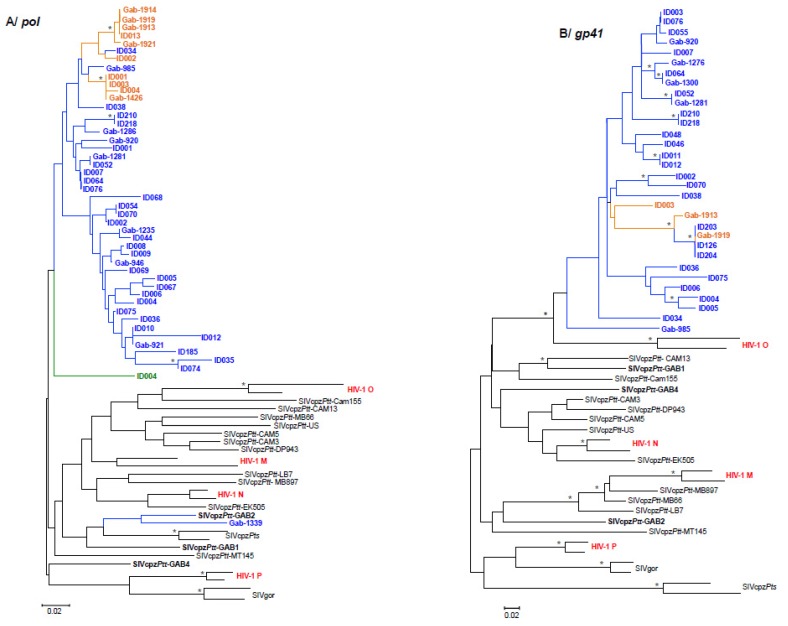
Phylogenetic analysis of partial *pol* (A) and *gp41* (B) of the newly identified SIVcpz sequences from Gabon. New partial *pol* (150bp) and *gp41* (170bp) SIVcpz-Gab sequences were compared to previously identified SIVcpz*Ptt* and SIVcpz*Pts* as well as HIV-1 goups M, N, O and P. Phylogenies were inferred using Neighbor-Joining method implemented in Mega 5 with the Kimura 2 Parameters model of evolution. Asterisks at nodes represent bootstraps values ≥70% (100 replicates). Scale bars indicate the number of base substitutions per site. New SIVcpz strains are colour-coded in accordance with figure 2B (MA in blue, ML in orange and IY in green). Strains amplified from genotyped animals are named using their ID number (IDXXX), whereas strains amplified from non-genotyped samples are named using the sample field number (GabXXXX). SIVcpz*Ptt*-Gab-1, -2 and -4 are shown in bold. HIV-1 groups M, N, O and P are shown in red.

Phylogenetic analyses ran on nucleotide fragments of 150 bp (*pol*) and of 170 bp (*gp41*) showed that all new SIVcpz*Ptt* strains obtained from the three sites (MA, IY and ML), formed a specific phylogeographic clade in both *pol* and *gp41* trees, within the SIVcpz*Ptt* cluster. Interestingly, these newly isolated SIVcpz*Ptt* strains were not closely related to the previously described SIVcpzGab-1, -2 and -4 except for SIVcpzGab1339, which clustered with SIVGab-2 in the *pol* phylogenetic tree (Figure 4).

**Figure 5:**
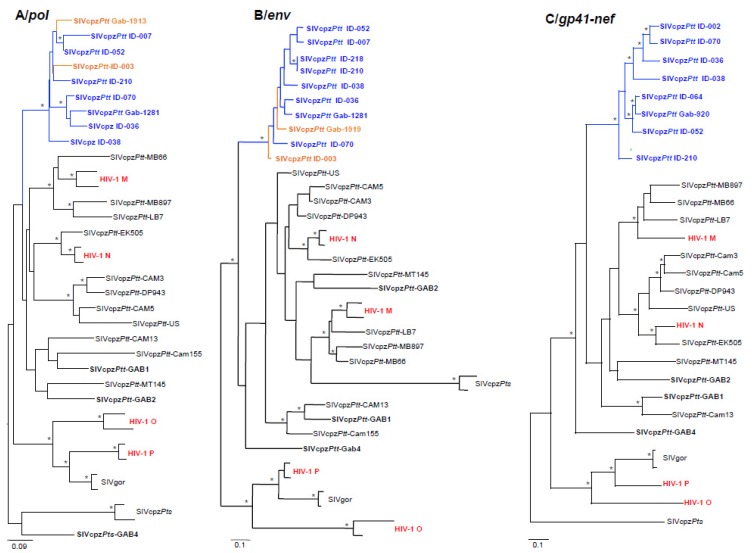
Phylogenetic analysis of partial *pol* (A), *env* (B) and *gp41-nef* (C) of the newly identified SIVcpz sequences from Gabon. New partial *pol* (365bp), *env (325bp)* and *gp41-nef* (916bp) SIVcpz-GAB sequences were compared to previously identified SIVcpz*Ptt* and SIVcpz*Pts* as well as HIV-1 goups M, N, O and P. Phylogenies were inferred using Maximum Likelihood methods implemented in PhyML under the GTR+Γ_4_+I model of evolution. Asterisks at nodes represent bootstrap values ≥70% (1000 replicates). Scale bars indicate the number of base substitutions per site. New SIVcpz strains are colour-coded in accordance with figure 2B (MA in blue and ML in orange). Strains amplified from genotyped animals are named using their ID number (IDXXX), whereas strains amplified from non-genotyped samples are named using the sample field number (GabXXXX). SIVcpz*Ptt*-Gab-1, -2 and -4 are shown in bold. HIV-1 groups M, N, O and P are shown in red.

Phylogenetic analyses ran on additional fragments of *pol*, *env* and *gp41-nef*, amplified in SIVcpz strains from MA and/or ML, showed that these newly obtained strains formed a highly supported clade (Figure 5). Moreover, all SIVcpz*Ptt* sequences from ML branched within the SIVcpz*Ptt* cluster of MA. Finally, the only SIVcpz strain (SIVcpzGab-2116-IYc-ID004) amplified from IY clustered at the root of the new SIVcpzGab clade in *pol* (Figure 4)*.* SIVcpz-Gab985 and 1033-ID034 clustered with all other SIVcpz strains from MA in *pol*, whereas they were at the root of the new SIVcpzGab clade in the *env* phylogenetic tree. We failed to amplify larger fragments for these three SIVcpz strains; therefore we were unable to draw any conclusion about their possible recombination structure.

### 3.3. Vertical and Horizontal Transmission of SIV within and between Chimpanzee Communities

#### 3.3.1. Allele Frequency Analysis

On 859 analyzed samples, 649 (75.5%) samples were successfully genotyped (Table 1). Allele frequency analysis on microsatellite results allowed us to identify 272 individuals (224-MA, 19-ML, 12-IY, 17-OD). Seven loci were typed, with a mean proportion of individuals typed of 0.97. Overall, the microsatellite loci used herein were highly polymorphic (mean polymorphic information content (PIC), 0.84), with a mean number of alleles per locus of 17.14, and an observed heterozygosity of 0.866. The goodness-of-fit test showed that all selected loci were at Hardy-Weinberg (HW) equilibrium except for locus D2S1326. A deviation from HW equilibrium at a single locus may occur because of natural selection on a nearby gene. It can also be an indicator of problems in genotyping that locus: e.g., a segregating null allele, a failure to consistently distinguish alleles, biases towards typing particular genotypes, *etc*. Ideally the locus should be excluded from analysis; in practice minor deviations from HW equilibrium at one or two loci are unlikely to significantly bias likelihoods calculated across all loci [41]. Cervus did not detect the presence of null alleles. All frequencies of null alleles we obtained were close to zero or slightly negative (negative values imply an excess of observed heterozygote genotypes). Locus D4S243 had a slightly positive frequency (+0.014). However, only with frequencies >0.05, it is advisable to exclude such a locus. 

#### 3.3.2. Simulation of Parentage Analysis

Simulations of parentage analysis reported that any candidate parent with a LOD score exceeding a critical value of 7.34 is assigned parentage with 95% confidence, and a LOD of 5.24 with a relaxed confidence of 80%.

#### 3.3.3. Parentage Analysis

Under a strict level of confidence (95%), 22% of the individuals included in the analysis were assigned a candidate parent (expected rate: 10%); under a relaxed level of confidence (80%), 51% of the individuals were assigned a candidate parent (expected rate: 23%). 

For the purpose of this study, only those individuals who share an SIV infection, detected by serology and/or PCR tests, and a parental link have been further discussed. We identified ten couples of individuals infected with SIV who shared a parental link at 95% confidence (Table S3). Among the individuals from the MA community, we found three pairs of males (ID-016 and ID-066; ID-031 and ID-066; ID-019 and ID-126), one pair of females (ID-003 and ID-077) and three male-female pairs (ID-059 and ID-063; ID-061 and ID-191; ID-132 and ID-210). Among the ML community we found three pairs of females sharing a parentage relation (ID-001 and ID-004; ID-003 and ID-006; ID-008). In addition, we also found a parental relation between a female from MA (ID-164) and a female from OD (ID-05). The female from OD did test negative for SIV. This is the only significant parental relationship that we found between communities.

A few of the above mentioned individuals had fragments of SIV successfully amplified. Phylogenetic trees of SIV *pol* and *gp41* fragments (Figure 4) and of SIV *pol*, *env*, and *gp41*/*nef* (Figure 5) from SIV infected individuals/samples showed that strains of viruses infecting chimpanzees inhabiting the MA region (depicted in blue) are interspersed with a few strains infecting chimpanzees from ML (depicted in orange). Unfortunately, we were not able to genotype a few samples, which SIV fragments are included in the trees. Among the individuals with a parentage relation, only ML individuals ID-001 and ID-004 and MA individuals ID-210 and ID-132 are represented in the phylogenetic trees. The individual ID-004 collected in IY (depicted in green in Figure 4) bears no parental relation with any other individual whose viruses are represented in phylogenetic trees.

## 4. Discussion

To our knowledge, this is the first report on SIV infection in wild-living chimpanzees (*Pan troglodytes troglodytes*) from Gabon. The SIV infection rate varied from 0% to 55% with an overall prevalence of 26.1%. Our data are consistent with previously documented rates from Cameroon and Tanzania [5,6]. In some locations, however, chimpanzees appeared not to be infected, as observed in some communities from Cameroon and Tanzania [5,6]. All the SIVcpz positive samples were collected north of the Ogooué River. To date, all SIVcpz positive samples isolated in west-central Africa have been found between the left bank of the Sanaga and the right bank of the Ogooué Rivers. The Ogooué River does not represent a genetic barrier for the subspecies of chimpanzee *Pan troglodytes troglodytes*, which is delimited instead by the Ubangi and the Congo River [21]. Little is known regarding the historical course and size of the Ogooué River, but rivers are known to change over time. Therefore, it is possible that at some point in time the river has lost (or acquired) the capacity of acting as a dispersal barrier. Exchange between chimpanzee populations might have been minimal at some point preventing SIV to cross the river [46,47,48]. Moreover, the relatively exiguous number of samples (*n* = 388) collected south of the Ogooué River does not allow us to be absolutely sure of the lack of SIV infection in chimpanzees living in these areas; additional studies are needed to confirm this.

In the north of Gabon, only one sample of 78 collected has a SIV positive serology result but the viral RNA could not be amplified (Figure 1). Of note, one of the first SIVcpz (SIVcpz-Gab-1) was identified in a chimpanzee captured in this region [27], suggesting that this virus was circulating among chimpanzee populations in the 1980s–1990s. However, the number of samples collected in this region was not sufficient and additional studies are needed to determine whether this virus is still circulating in this area and at which prevalence.

In the northeast of Gabon instead, all the chimpanzee communities on the left bank of the Ivindo River were infected with SIVcpz. These animals live in a continuous forest block, delimited westward by the Ivindo, southward by the Ogooué River (Figure 1) and to the east by the Republic of Congo with the Sangha River as a principal natural barrier. The collection sites, except for Djidji (DJ), which is situated inside the Ivindo National Park on the left bank of the Ivindo River, are adjacent to the villages of Odjala (OD), Makatamangoye 2 (MA), Iyoko milieu (IY) and Malouma (ML) (Figure 3). Taking into account the number of individuals identified, the prevalence observed was 46% for MA and 63.5% for ML. However, the prevalence rate observed in ML was likely overestimated since we failed to obtain genotype in half of the samples. We found some discordance in the serology results, with some individuals being first sero-positive followed by samples, which became sero-negative. The lack of sensitivity of the serology test may be due to: (I) the presence of Antigen-antibody binding inhibitors in the faecal samples [49]; (II) the collection of a faecal sample later in the day, which could contain a lower titer of antibodies compared to the first morning defecation; (III) the degradation of the faecal material.

Besides, we obtained SIV RNA for only 23.4% of the specimens, which resulted SIV positive by INNO-LIA I/II score Confirmation test. This result could be due to either a low sensitivity of the molecular tools or a low viral load in faeces or to the natural clearance of the virus in the host. *A contrario*, in randomly selected SIV antibodies negative samples from MA, we succeeded in amplifying SIV fragments in 14% of them (Table 1). Altogether, these results suggest that we may have underestimated the SIV prevalence rate. It also highlights the importance of developing new serological and molecular tools for viral detection and characterization in faecal samples. A census made of chimpanzee populations throughout Gabon between December 1980 and February 1983 estimated 64,000 ± 13,000 animals living in the country [50]. This population has since decreased in number and density, due to Ebola virus outbreaks, diminished resources and habitat and human pressure and hunting [51]. Because it is difficult to follow non-habituated chimpanzees in the wild and collect consecutive samples, non-invasive sampling remains the preferred approach for these studies.

The phylogenetic analyses showed that the different SIV fragments amplified in *pol* and/or *env*, obtained from chimpanzees from MA, IY, and ML cluster together in phylogenetic trees, but are separate from the strains of SIVcpz*Ptt* previously isolated in Cameroon and in Gabon (Figure 4 and Figure 5). In fact, the position in the phylogeny of SIVcpz*Ptt*-Gab-1, -2 and -4 varies according to the gene investigated. SIVcpz*Ptt*-Gab-1, -2 and -4 are not linked to the new strains identified in this study, except for SIVcpz-Gab1339, isolated in a chimpanzee collected in MA, which gene *pol* was partially amplified. SIVcpz-Gab1339 is tightly linked to SIVcpz-Gab-2, isolated in a chimpanzee captured 30 years ago around the same village. Unfortunately, we were not able to amplify a larger fragment of its genome to study more in detail the phylogenetic similarities of the two strains. It is however surprising that only one of the strains partially amplified from MA is phylogenetically close to SIVcpz-Gab-2. Further research, i.e. the amplification of the full-length genome of SIVcpz-Gab1339 and of other strains circulating in that area is needed to know whether this new strain is the result of the recombination of old and extant strains, or whether SIVcpz-Gab-2 was a viral strain weakly represented or supplanted by the introduction of a new SIV in these chimpanzee communities. Of note, phylogenetic analyses of SIVcpz*Ptt-*Gab-2 full-length genome suggested that this strain itself was the result of past recombination events [52].

Our results also demonstrated that there might be an ongoing viral exchange between communities. It is worth noting that, during the three field trips conducted in ML, we encountered only one group of chimpanzees estimated to be composed of about 30 to 40 adult individuals (adults and juveniles). This village is situated in an area, which has been hardly hit by several Ebola outbreaks in the early years 2000. This epidemic led to a catastrophic ape decline in Gabon and, more in general, in western equatorial Africa [53,54,55]. The genetic similarity between the SIV infecting this community and the SIV infecting chimpanzees from Makatamangoye 2 may be the result of the migration of infected chimpanzees from the south to the northern region of Malouma. We also showed that female chimpanzees can migrate from or to regions that are about 30 km apart, as reported for the mother-daughter pair ID-164 (MA) and ID-005 (OD). The phylogenetic trees analyses (Figure 4 and Figure 5) showed closely related viruses that suggested potential epidemiologic links between the infected animals. Unrelated individuals sharing very similar viruses, demonstrate the spread of SIV via horizontal routes (due to sexual or aggressive behaviors, depending on the sexes involved). Related individuals like the MA pair male/female and the female/female pair from ML are testimony of possible vertical transmissions of the virus. A few more related individuals have been identified, if we consider the serology results as well, but because we do not have any molecular details about these strains, we cannot speculate about the viral transmission routes involved.

Therefore, in order to better understand the spread dynamic of SIV within and between ape’s communities, it would be necessary to complete the host genetic profiles from infected and non-infected individuals, to amplify SIV fragments from more samples that tested positive for Antibodies, and to complete genomes.

Altogether, our data showed that there is a high prevalence of SIV infection in chimpanzee communities living in the northeast of Gabon and a high genetic diversity of the circulating SIVcpz strains with at least four distinct clades. Previous studies showed that SIV infection in eastern chimpanzees had a negative impact on their health and reproduction [12]. In central Africa, only wild born chimpanzee held captive showed signs of an AIDS-like disease [13,14]. Thus, the impact of such infection in wild chimpanzees from west central Africa needs to be further evaluated.

The human populations living along the Odjala-Malouma road axe have access to the forest, and bushmeat is their main source of animal proteins. Chimpanzees are hunted for meat, although in smaller proportions compared to other mammals [56]. Given the high prevalence of SIV detected in the region, the risk of transmission to the human population should not be ignored. In fact, this region represents a hot-spot for a potential emergence of a new Human Immunodeficiency Virus.

## 5. Conclusion

Our work showed for the first time a high rate of SIVcpz infection in wild-living chimpanzee communities from northeastern Gabon. Previous studies, led by our research team, discovered the origin of the four HIV-1 groups known to date from chimpanzees and gorillas inhabiting the Cameroonian rain forest [1,2,3,4,7]. The majority of faecal samples analyzed in this study have been collected less than 20 km away from villages. This area of Gabon is relatively remote and people depend on forest resources for living. Although it is difficult to estimate the number of chimpanzees hunted per year, the informal discussions we had with the villagers suggested that this practice is not rare. In this context, the establishment of sentinel surveillance among human populations would be helpful to anticipate the potential emergence of a new Human Immunodeficiency Virus.

## Supplementary Materials

Supplementary materials can be found at: http://www.mdpi.com/1999-4915/7/9/2855/s1.
