# Supplementary material for: High Rate of Simian Immunodeficiency Virus (SIV) Infections in Wild Chimpanzees in Northeastern Gabon
Source: Viruses. 2015 Sep 15;7(9):4997–5015. doi: 10.3390/v7092855 (PMC4584299; doi:10.3390/v7092855)
Supplement: Supplementary file 1 [file viruses-07-02855-s001.pdf]

Supplementary Information

Table S1. SIV analysis results and consensus genetic profile of chimpanzees collected in MA, ML, IY and OD.

Individual MA site	Lab Code	Fecal Antibody Detection	RT-PCR		Sampling		Loci						
			pol	*gp41*	Date	Sex	D18S536	D4S243	D10S676	D9S922	D2S1326	D2S1333	D4S1627
MA-ID-001	Gab-0904	+	+	−	07/04/11	F	150/162	225/229	179/183	274/298	235/247	318/322	215/235
MA-ID-002	Gab-0908	+	+	+	12/04/11	M	162/178	225/237	183/187	290/302	235/243	306/322	231/235
	Gab-0950	+	+	+	13/04/11		162/178	225/237	- / -	- / -	235/243	- / -	231/235
MA-ID-003	Gab-0917	+	−	+	14/04/11	F	150/170	197/225	183/195	278/294	247/255	- / -	211/227
MA-ID-004	Gab-0928	-	+	+	08/04/11	F	154/170	225/229	175/179	286/298	231/235	314/322	211/223
MA-ID-005	Gab-0938	+	+	+	11/04/11	F	154/174	225/229	179/179	302/310	235/255	306/318	211/243
MA-ID-006	Gab-0942	+	+	+	11/04/11	M	158/166	225/229	183/183	274/298	243/255	306/310	215/215
MA-ID-007	Gab-0944	+	+	+	11/04/11	M	138/162	221/225	167/187	274/302	227/231	310/322	- / -
MA-ID-008	Gab-0945	+	+	−	14/04/11	M	162/166	225/237	175/183	282/298	231/247	306/314	191/219
MA-ID-009	Gab-0947	+	+	−	14/04/11	M	150/170	225/229	167/183	302/306	227/243	306/322	211/219
MA-ID-010	Gab-0902	-	−	−	07/04/11	F	170/182	225/229	179/183	302/302	231/235	306/306	211/223
	Gab-1001	+	+	−	17/05/11		170/182	225/229	179/183	302/302	231/235	306/306	211/223
MA-ID-011	Gab-1017	+	−	+	20/05/11	F	166/170	225/245	179/187	274/294	227/251	318/322	191/223
MA-ID-012	Gab-1023	+	+	+	20/05/11	F	146/170	225/245	167/167	294/302	231/255	306/330	219/227
MA-ID-013	Gab-0913	+	−	−	13/04/11	M	150/182	225/245	179/183	294/298	235/235	310/322	211/235
MA-ID-014	Gab-0962	+	−	−	07/04/11	F	150/174	229/245	179/183	290/290	235/247	302/322	231/243
	Gab-0963	+	−	−	07/04/11		150/174	229/245	179/183	290/290	235/247	302/322	231/243
	Gab-0964	+	−	−	07/04/11		150/174	229/245	179/183	290/290	235/247	302/322	231/243
	Gab-0965	+	−	−	07/04/11		150/174	229/245	179/183	290/290	235/247	302/322	231/243
MA-ID-015	Gab-0995	+	−	−	04/05/11	F	150/178	201/253	167/179	250/274	183/219	230/234	203/219
MA-ID-016	Gab-1000	+	−	−	17/05/11	M	134/178	217/245	171/179	242/298	223/231	306/314	219/227
	Gab-1004	+	−	−	17/05/11		134/178	217/245	171/179	242/298	223/231	306/314	219/227
	Gab-1006	+	−	−	17/05/11		134/178	217/245	171/179	242/298	223/231	306/314	219/227

Table S1. *Cont.*

Individual MA site	Lab Code	Fecal Antibody Detection	RT-PCR		Sampling		Loci						
			pol	*gp41*	Date	Sex	D18S536	D4S243	D10S676	D9S922	D2S1326	D2S1333	D4S1627
MA-ID-017	Gab-1003	+	−	−	17/05/11	M	134/154	189/197	167/183	278/294	223/231	306/314	211/235
MA-ID-018	Gab-1005	+	−	−	17/05/11	M	170/190	221/257	179/183	250/290	231/235	- / -	- / -
MA-ID-019	Gab-1013	+	−	−	20/05/11	M	166/178	225/253	179/187	250/302	227/235	306/306	211/219
MA-ID-020	Gab-1031	+	−	−	07/06/11	M	146/170	217/229	171/183	302/306	223/243	306/314	219/227
MA-ID-021	Gab-0907	+	−	−	11/04/11	M	154/158	205/237	175/179	286/294	231/231	306	227/231
MA-ID-022	Gab-0910	+	−	−	13/04/11	F	134/174	221/221	179/187	286/298	227/231	310/326	219/219
MA-ID-023	Gab-0949	+	−	−	14/04/11	M	146/170	221/245	179/179	294/298	231/235	314/318	219/223
	Gab-1500	+	−	−	08/03/12		146/170	221/245	179/179	294/298	231/235	314/318	219/223
MA-ID-024	Gab-0957	−	−	−	14/04/11	M	154/166	225/225	163/167	290/290	235/247	314/322	231/231
	Gab-0959	+	−	−	14/04/11		154/166	225/225	167/167	- / -	235/247	314/322	231/231
MA-ID-025	Gab-0977	+	−	−	02/05/11	F	170/186	233/233	179/183	274/298	247/255	314/322	227/243
	Gab-0978	+	−	−	02/05/11		170/186	233/233	179/183	274/298	247/255	314/322	227/243
	Gab-0981	−	−	−	02/05/11		170/186	233/233	179/183	274/298	247/255	314/322	227/243
MA-ID-026	Gab-0997	+	−	−	17/05/11	F	150/162	193/229	175/183	298/314	227/231	306/306	227/235
MA-ID-027	Gab-0999	+	−	−	17/05/11	M	158/170	233/241	179/183	294/298	231/235	302/318	211/211
	Gab-1019	+	−	−	20/05/11		158/170	233/241	179/183	294/298	231/235	302/318	211/211
MA-ID-028	Gab-1012	+	−	−	20/05/11	M	138/170	193/229	179/183	290/302	227/235	318/322	235/235
	Gab-2092	−	/	/	08/07/12		138/170	193/229	179/183	290/302	227/235	318/322	235/235
MA-ID-029	Gab-1014	+	−	−	20/05/11	M	158/170	- / -	167/183	294/302	231/255	306/318	211/227
MA-ID-030	Gab-1015	+	−	−	20/05/11	M	166/190	225/245	179/183	298/302	223/231	310/314	211/223
MA-ID-031	Gab-1020	+	-	-	20/05/11	M	134/170	217/229	171/179	274/298	223/231	- / -	219/227
MA-ID-032	Gab-1021	+	-	-	20/05/11	F	146/158	- /229	183/239	290/302	231/255	302/322	239/243
	Gab-1027	+	-	-	20/05/11		146/158	- /229	183/239	290/302	231/255	302/322	239/243
	Gab-2457	+	/	/	08/05/13		- / -	- / -	183/ -	290/ -	231/255	302/322	239/243
	Gab-2532	+	/	/	11/05/13		146/158	201/229	183/239	290/302	231/255	302/322	239/243
	Gab-2535	+	/	/	11/05/13		146/158	201/229	183/239	290/302	231/255	302/322	239/243

Table S1. *Cont.*

Individual MA site	Lab Code	Fecal Antibody Detection	RT-PCR *pol*	RT-PCR *gp41*	Sampling Date	Sampling Sex	Loci D18S536	D4S243	D10S676	D9S922	D2S1326	D2S1333	D4S1627
MA-ID-033	Gab-1025	+	−	−	20/05/11	F	170/194	225/277	179/179	250/286	235/243	318/322	219/219
MA-ID-034	Gab-1033	−	+	+	07/06/11	M	142/150	217/233	179/179	290/298	219/235	302/306	231/239
MA-ID-035	Gab-1220	+	+	−	28/10/11	M	166/166	221/229	175/179	274/294	227/243	310/330	231/243
	Gab-1221	+	−	−	28/10/11		166/ -	221/ -	175/179	274/294	227/243	310/330	231/243
	Gab-1222	+	−	−	28/10/11		166/166	221/229	175/179	274/294	227/243	310/330	231/243
MA-ID-036	Gab-1227	+	+	+	31/10/11	F	134/166	225/233	167/183	290/306	231/235	306/322	231/ -
	Gab-1228	+	+	+	31/10/11		134/166	225/233	167/183	290/306	231/235	306/322	231/239
	Gab-1229	+	+	+	31/10/11		134/166	225/233	167/183	- / -	231/235	- /322	- / -
	Gab-1230	+	+	+	31/10/11		134/166	225/233	167/183	290/306	231/235	306/322	231/239
	Gab-1510	+	−	−	11/03/12		134/166	225/233	167/183	290/306	231/235	306/322	231/239
	Gab-1513	+	−	−	11/03/12		134/166	225/233	167/183	290/306	- /235	306/322	231/239
	Gab-1515	−	/	/	11/03/12		134/166	225/233	167/183	290/ -	- / -	306/ -	- / -
	Gab-1517	+	−	−	11/03/12		134/166	225/233	167/183	290/306	231/235	306/322	231/239
	Gab-1518	+	−	−	11/03/12		134/166	225/233	167/183	290/306	231/235	306/322	231/239
MA-ID-037	Gab-1184	+	−	−	24/10/11	M	138/138	229/245	167/179	298/302	235/247	306/310	215/227
	Gab-1185	+	−	−	24/10/11		138/138	229/245	167	298/302	235/247	306/310	215/227
	Gab-1232	+	−	−	09/11/11		138/138	229/245	167	298/302	235/247	306/310	215/227
	Gab-1497	+	−	−	08/03/12		138/138	229/245	167/179	298/302	235/247	306/310	215/227
MA-ID-038	Gab-1253	+	+	+	06/11/11	M	158/170	209/221	171/183	294/294	231/243	310/322	219/223
	Gab-2266	+	−	−	17/12/13		158/170	209/221	171/183	294/294	231/243	310/322	219/223
	Gab-2282	+	/	/	19/02/13		158/170	209/221	171/183	294/294	231/243	310/322	219/223
	Gab-2307	+	+	+	04/03/13		158/170	209/221	171/183	294/294	231/243	310/322	219/223
	Gab-2322	+	−	−	04/03/13		158/170	209/221	171/183	294/294	231/243	310/322	219/223
	Gab-2324	+	−	+	04/03/13		158/170	209/221	171/183	294/294	231/243	310/322	219/223
MA-ID-039	Gab-1212	+	−	−	17/10/11	F	158/166	225/225	167/179	286/294	231/255	306/330	219/243
	Gab-1213	+	−	−	17/10/11		158/166	225/225	167/179	286/294	231/255	306/330	219/243

Table S1. *Cont.*

Individual MA site	Lab Code	Fecal Antibody Detection	RT-PCR		Sampling		Loci						
			pol	*gp41*	Date	Sex	D18S536	D4S243	D10S676	D9S922	D2S1326	D2S1333	D4S1627
MA-ID-040	Gab-1214	+	−	−	17/10/11	M	150/170	225/245	175/179	274/302	231/243	310/326	227/243
	Gab-1215	+	−	−	17/10/11		150/170	225/245	175/179	274/302	231/243	310/326	227/243
	Gab-1216	+	−	−	17/10/11		150/170	225/245	175/179	274/302	231/243	310/326	227/243
	Gab-1217	+	−	−	17/10/11		150/170	225/245	175/179	274/302	231/243	310/326	227/243
	Gab-1218	+	−	−	17/10/11		150/170	225/245	175/179	274/302	231/243	310/326	227/243
MA-ID-041	Gab-1233	+	−	−	18/10/11	M	146/166	185/245	179/179	294/310	231/255	310/314	211/223
	Gab-1473	+	−	−	09/03/12		146/166	185/245	179/179	294/310	231/255	310/314	211/223
	Gab-1491	+	−	−	09/03/12		146/166	185/245	179/179	294/310	231/255	310/314	211/223
MA-ID-042	Gab-1263	+	−	−	03/11/11	M	150/150	209/245	175/179	290/302	235/255	314/326	219/223
	Gab-1264	+	−	−	03/11/11		150/150	209/245	175/179	290/302	235/255	314/326	219/223
MA-ID-043	Gab-1267	+	−	−	26/10/11	M	166/170	193/221	175/183	302/306	223/231	318/326	231/255
MA-ID-044	Gab-1275	+	+	−	01/12/11	M	134/170	201/225	167/179	294/298	- /235	310/318	211/239
	Gab-1302	−	/	/	02/12/11		134/170	201/225	167/179	294/298	235/235	310/318	211/239
	Gab-1303	−	/	/	02/12/11		134/170	201/225	167/179	294/298	235/235	310/318	211/239
MA-ID-045	Gab-1176	+	−	−	24/10/11	M	150/170	193/201	171/179	298/298	227/231	306/322	219/243
	Gab-1177	+	−	−	24/10/11		150/170	193/201	171/179	298/298	227/231	306/322	219/ -
	Gab-1476	+	−	−	10/03/12		150/170	193/201	171/179	298/298	227/231	306/322	219/243
	Gab-1502	+	−	−	08/03/12		150/170	193/201	171/179	298/298	227/231	306/322	219/243
MA-ID-046	Gab-1266	+	−	+	26/09/11	M	154/170	- /225	175/175	298/302	231/235	306/326	211/223
MA-ID-047	Gab-2530	−	/	/	09/05/13	M	174/178	217/225	183/183	298/302	231/235	306/306	219/223
MA-ID-048	Gab-1239	+	−	+	21/10/11	F	134/170	205/225	179/183	294/298	255/263	314/322	219/223
MA-ID-049	Gab-1240	+	−	−	21/10/11	M	150/170	201/225	167/167	294/310	251/255	306/310	215/231
MA-ID-050	Gab-1241	+	−	−	21/10/11	M	150/158	- /229	183/187	286/290	215/231	306/314	215/231
	Gab-1242	+	−	−	21/10/11		150/158	225/229	183/187	286/290	215/231	306/314	215/231
MA-ID-051	Gab-1279	+	−	−	01/12/11	M	150/158	201/245	167/183	290/294	231/235	306/306	223/231
	Gab-1280	+	−	−	01/12/11		150/158	201/245	167/183	290/294	231/235	306/306	223/231

Table S1. *Cont.*

Individual MA site	Lab Code	Fecal Antibody Detection	RT-PCR		Sampling		Loci						
			pol	*gp41*	Date	Sex	D18S536	D4S243	D10S676	D9S922	D2S1326	D2S1333	D4S1627
MA-ID-052	Gab-1282	+	+	−	01/12/11	M	150/182	205/229	179/179	298/302	231/235	306/318	215/235
	Gab-1284	+	+	+	01/12/11		150/182	205/229	179/179	298/302	231/235	306/318	215/235
	Gab-1287	+	+	+	01/12/11		150/182	205/229	179/179	298/302	231/235	306/318	215/235
	Gab-2096	+	−	−	07/12/12		150/182	205/229	179/179	- /302	231/235	306/318	215/235
MA-ID-053	Gab-1285	+	−	−	01/12/11	F	134/166	221/225	179/183	290/294	227/255	310/334	191/239
	Gab-1291	+	−	−	01/12/11		134/166	221/225	179/183	290/294	227/255	310/334	191/239
MA-ID-054	Gab-0923	−	−	−	07/04/11	M	150/154	221/241	179/187	274/302	239/259	310/314	227/231
	Gab-0924	−	−	−	07/04/11		150/154	221/241	179/187	274/302	239/259	310/314	227/231
	Gab-1283	+	−	−	01/12/11		150/154	221/241	179/187	274/302	239/259	310/314	227/231
	Gab-1288	+	−	−	01/12/11		150/154	221/241	179/187	274/302	239/259	310/314	227/231
	Gab-1289	+	−	−	01/12/11		150/154	221/241	179/187	274/302	239/259	310/314	227/231
	Gab-1295	+	+	−	01/12/11		150/154	221/241	179/187	274/302	239/259	310/314	227/231
	Gab-1321	+	−	−	03/12/11		150/154	221/241	179/187	274/302	239/259	310/314	227/231
MA-ID-055	Gab-0926	−	−	−	07/04/11	M	150/162	221/245	183/183	298/310	231/235	310/322	239/243
	Gab-1293	+	−	−	01/12/11		150/162	221/245	183/183	298/310	231/235	310/322	239/243
	Gab-1294	+	−	+	01/12/11		150/162	221/245	183/183	298/310	231/235	310/322	239/243
MA-ID-056	Gab-0922	−	−	−	07/04/11	F	170/ -	229/ -	167/179	298/302	231/ -	306/322	219/239
	Gab-1297	+	−	−	01/12/11		170/182	229/237	167/179	298/302	231/243	306/322	219/239
MA-ID-057	Gab-1306	+	−	−	02/12/11	F	150/154	193/225	175/179	294/298	231/267	306/322	219/227
	Gab-1307	+	−	−	02/12/11		150/154	193/225	175/179	294/298	231/267	306/322	219/227
	Gab-1308	+	−	−	02/12/11		150/154	193/225	175/179	294/298	231/267	306/322	219/227
	Gab-1309	+	−	−	02/12/11		150/154	193/225	175/179	294/298	231/267	306/322	219/227
	Gab-1310	+	−	−	02/12/11		150/154	193/225	175/179	294/298	231/267	306/322	219/227
MA-ID-058	Gab-0915	−	−	−	07/04/11	F	150/154	229/229	175/179	290/298	231/235	306/314	223/235
	Gab-0929	−	−	−	07/04/11		150/154	229/229	175/179	290/298	231/235	306/314	223/235
	Gab-1314	+	−	−	02/12/11		150/154	229/229	175/179	290/298	231/235	306/314	223/235

Table S1. *Cont.*

Individual MA site	Lab Code	Fecal Antibody Detection	RT-PCR *pol*	RT-PCR *gp41*	Sampling Date	Sampling Sex	Loci D18S536	D4S243	D10S676	D9S922	D2S1326	D2S1333	D4S1627
	Gab-2488	+	/	/	06/05/13		150/154	229/229	175/179	290/298	231/235	306/314	223/235
	Gab-2489	+	/	/	06/05/13		150/154	229/229	175/179	290/298	231/235	306/314	223/235
MA-ID-059	Gab-1315	+	−	−	02/12/11	M	134/170	225/225	175/179	298/302	235/243	318/330	227/239
MA-ID-060	Gab-1187	+	−	−	14/10/11	M	154/170	225/229	175/179	274/302	235/255	306/322	223/231
	Gab-1471	+	−	−	09/03/12		154/170	225/229	175/179	274/302	235/255	306/322	223/231
	Gab-1484	+	−	−	08/03/12		154/170	225/229	175/ -	274/302	235/255	306/ -	223/231
	Gab-1490	+	−	−	08/03/12		154/170	225/229	175/179	274/ -	235/255	306/322	223/231
	Gab-1493	+	−	−	08/03/12		154/170	225/229	175/179	274/302	235/255	306/322	223/231
MA-ID-061	Gab-1248	+	−	−	06/11/11	F	126/146	229/245	183/183	290/290	227/235	314/326	219/235
	Gab-1255	+	−	−	06/11/11		126/146	229/245	183/183	290/290	227/235	314/326	219/235
	Gab-1259	+	−	−	06/11/11		126/146	229/245	183/183	290/290	227/235	314/326	219/235
	Gab-2276	+	/	/	19/02/13		126/146	229/245	183/183	290/290	227/235	314/326	219/235
	Gab-2279	+	/	/	19/02/13		126/146	229/245	183/183	290/290	227/235	314/326	219/235
	Gab-2280	+	/	/	19/02/13		126/146	229/245	183/183	290/290	227/235	314/326	219/235
	Gab-2281	+	/	/	19/02/13		126/146	229/245	183/183	290/290	227/235	314/326	219/235
	Gab-2288	+	/	/	19/02/13		126/146	229/245	- /183	- /290	227/235	314/326	219/235
	Gab-2296	+	/	/	19/02/13		126/146	229/ -	183/183	290/290	227/235	314/326	219/235
	Gab-2299	+	/	/	19/02/13		126/146	229/ -	183/183	290/290	227/235	314/326	219/235
	Gab-2302	+	/	/	19/02/13		126/146	229/ -	183/183	290/290	227/235	314/326	219/235
	Gab-2303	+	/	/	19/02/13		126/146	229/245	183/183	- /290	227/235	314/326	219/235
MA-ID-062	Gab-1316	+	−	−	02/12/11	M	150/154	225/229	175/179	298/310	231/239	306/330	223/223
	Gab-1317	+	−	−	02/12/11		150/154	225/229	175/179	298/310	231/239	306/330	223/223
MA-ID-063	Gab-1311	+	−	−	02/12/11	F	166/170	225/225	175/179	298/302	243/247	326/330	227/239
	Gab-1312	+	−	−	02/12/11		166/170	225/225	175/179	298/302	243/247	326/330	227/239
MA-ID-064	Gab-1320	+	−	+	03/12/11	M	150/186	225/245	179/183	294/298	231/235	310/322	211/211
MA-ID-065	Gab-1336	+	−	−	01/12/11	M	158/170	201/209	167/183	294/306	223/231	306/318	219/227

Table S1. *Cont.*

Individual MA site	Lab Code	Fecal Antibody Detection	RT-PCR		Sampling		Loci						
			pol	*gp41*	Date	Sex	D18S536	D4S243	D10S676	D9S922	D2S1326	D2S1333	D4S1627
	Gab-1337	+	−	−	01/12/11		158/170	201/209	167/183	294/306	223/231	306/318	219/227
	Gab-1963	+	−	−	26/10/12		158/170	201/209	167/183	294/306	223/231	306/318	219/227
MA-ID-066	Gab-1338	+	−	−	02/12/11	M	134/182	217/229	171/179	298/298	223/231	306/314	219/227
MA-ID-067	Gab-1349	+	−	−	03/12/11	M	138/170	221/245	167/179	274/290	227/251	306/310	191/227
	Gab-1351	+	−	−	03/12/11		138/170	221/245	167/179	274/290	227/251	306/310	191/227
	Gab-1352	+	+	−	03/12/11		138/170	221/245	167/179	274/290	227/251	306/310	191/227
	Gab-1353	+	−	−	03/12/11		138/170	221/245	167/179	274/290	227/251	306/310	191/227
	Gab-1354	+	−	−	03/12/11		138/170	221/245	167/179	274/290	227/251	306/310	191/227
	Gab-1355	+	−	−	03/12/11		138/170	221/245	167/179	274/290	227/251	306/310	191/227
	Gab-1356	+	−	−	03/12/11		138/170	221/245	167/179	274/290	227/251	306/310	191/227
MA-ID-068	Gab-1367	+	−	−	08/12/11	F	162/170	197/225	175/179	294/306	231/235	306/306	223/227
	Gab-1369	+	+	−	08/12/11		162/170	197/225	175/179	294/306	231/235	306/306	223/227
	Gab-1371	+	−	−	08/12/11		162/170	197/225	175/179	294/306	231/235	306/306	223/227
	Gab-1373	+	−	−	08/12/11		162/170	197/225	175/179	294/306	231/235	306/306	223/227
	Gab-1374	+	−	−	08/12/11		162/170	197/225	175/179	294/306	231/235	306/306	223/227
MA-ID-069	Gab-1472	+	+	−	09/03/12	F	146/158	201/225	175/179	294/306	231/235	310/322	211/239
MA-ID-070	Gab-1290	+	+	+	02/12/11	M	158/170	185/249	183/183	290/302	243/243	314/318	219/231
	Gab-1292	+	+	+	02/12/11		158/170	185/249	183/183	290/302	243/243	314/318	219/231
MA-ID-071	Gab-1350	+	−	−	03/12/11	M	138/150	221/229	175/179	286/314	243/251	306/310	223/227
	Gab-1357	−	/	/	03/12/11		138/150	221/229	175/179	286/314	243/251	306/310	223/227
	Gab-1358	−	/	/	03/12/11		138/150	221/229	175/179	286/314	243/251	306/310	223/227
	Gab-1359	−	/	/	03/12/11		138/150	221/229	175/179	286/314	243/251	306/310	223/227
	Gab-1360	−	/	/	03/12/11		138/150	221/229	175/179	286/314	243/251	306/310	223/227
	Gab-1361	−	/	/	03/12/11		138/150	221/229	175/179	286/314	243/251	306/310	223/227
	Gab-1362	−	/	/	03/12/11		138/150	221/229	175/179	286/314	243/251	306/310	223/227
	Gab-1363	−	/	/	03/12/11		138/150	221/229	175/179	286/314	243/251	306/310	223/227

Table S1. *Cont.*

Individual MA site	Lab Code	Fecal Antibody Detection	RT-PCR		Sampling		Loci						
			pol	*gp41*	Date	Sex	D18S536	D4S243	D10S676	D9S922	D2S1326	D2S1333	D4S1627
	Gab-1364	−	/	/	03/12/11		138/150	221/229	175/179	286/314	243/251	306/310	223/227
MA-ID-072	Gab-1474	+	−	−	09/03/12	F	138/170	229/229	179/183	290/306	239/243	306/314	211/227
MA-ID-073	Gab-1503	+	−	−	08/03/12	F	170/170	193/197	179/183	298/306	239/255	306/310	219/235
	Gab-1504	+	−	−	08/03/12		170/170	193/197	179/183	298/306	239/255	306/310	219/235
	Gab-1507	+	−	−	08/03/12		170/170	193/197	179/183	298/306	239/255	306/310	219/235
MA-ID-074	Gab-1209	+	−	−	17/10/11	M	150/166	225/225	167/179	294/302	- / -	306/310	219/243
	Gab-1210	+	−	−	17/10/11		150/166	225/225	167/179	294/302	- / -	306/310	- /243
	Gab-1211	+	+	−	17/10/11		150/166	225/225	167/179	294/302	235/255	306/310	219/243
MA-ID-075	Gab-1296	−	+	+	02/12/11	M	146/182	225/225	183/183	290/298	235/243	322/322	211/235
MA-ID-076	Gab-1298	+	−	+	02/12/11	M	150/170	205/225	167/183	- /302	235/267	306/310	235/239
	Gab-1319	+	+	+	03/12/11		150/170	205/225	167/183	290/302	235/267	306/310	239/239
MA-ID-077	Gab-0919	−	−	−	07/04/11	F	150/170	201/225	- / -	- /294	247/255	322/322	211/227
	Gab-1301	+	−	−	02/12/11		150/170	201/225	183/195	294/294	247/255	322/322	211/227
MA-ID-078	Gab-2366	−	+	+	03/03/13	M	134/138	225/245	- /179	306/306	227/231	326/326	219/223
	Gab-2446	−	/	/	04/04/13		134/138	225/245	175/179	306/306	227/231	326/326	219/223
	Gab-2453	−	/	/	11/04/13		134/138	225/245	175/179	306/306	227/231	326/326	219/223
MA-ID-079	Gab-1188	−	/	/	24/10/11	M	138/158	185/193	183/183	298/298	231/235	306/322	211/227
	Gab-1231	−	/	/	08/11/11		138/158	185/193	183/183	298/298	231/235	306/322	211/227
MA-ID-080	Gab-1972	−	/	/	10/11/12	F	142/146	193/201	179/183	294/298	223/243	314/318	231/235
	Gab-1975	−	/	/	10/11/12		- /146	193/201	- /183	294/298	223/243	314/318	231/235
MA-ID-081	Gab-0905	+	−	−	08/04/11	F	154/158	225/225	167/183	286/302	235/243	310/314	227/ -
	Gab-0989	−	−	−	04/05/11		154/158	225/225	167/183	286/302	235/243	310/314	- / -
	Gab-1010	−	−	−	20/05/11		154/158	225/225	167/183	286/302	235/243	310/314	227/247
	Gab-1011	−	−	−	20/05/11		154/158	225/225	167/183	286/302	235/243	310/314	227/247
	Gab-1016	+	−	−	20/05/11		154/158	- /225	167/183	286/302	235/243	310/314	227/247
	Gab-1018	−	−	−	20/05/11		154/158	225/225	167/183	286/302	235/243	310/314	227/247

Table S1. *Cont.*

Individual MA site	Lab Code	Fecal Antibody Detection	RT-PCR *pol*	RT-PCR *gp41*	Sampling Date	Sampling Sex	Loci D18S536	D4S243	D10S676	D9S922	D2S1326	D2S1333	D4S1627
	Gab-2486	−	/	/	06/05/13		154/158	225/225	167/183	286/302	235/243	310/314	227/247
MA-ID-082	Gab-1331	−	/	/	02/12/11	F	174/178	205/245	167/179	310/318	235/251	306/306	211/219
	Gab-2531	−	/	/	11/05/13		174/178	205/245	167/179	- / -	235/251	306/306	211/219
MA-ID-083	Gab-0906	−	−	−	08/04/11	F	170/194	245/245	179/183	298/306	231/255	326/330	211/223
MA-ID-084	Gab-0912	−	−	−	13/04/11	M	158/162	225/225	175/187	302/302	231/235	306/322	231/235
	Gab-1304	−	/	/	02/12/11		158/162	225/225	175/187	302/302	231/235	306/322	231/235
MA-ID-085	Gab-0914	−	−	−	13/04/11	F	134/166	201/221	175/179	294/294	231/235	306/310	211/219
MA-ID-086	Gab-2528	−	/	/	09/05/13	M	170/178	225/241	183/183	290/302	235/251	306/318	211/247
MA-ID-087	Gab-2526	−	/	/	09/05/13	F	158/162	225/245	179/183	294/302	231/235	306/318	191/219
MA-ID-088	Gab-0925	−	−	−	07/04/11	F	150/170	229/245	179/183	298/302	231/235	306/310	239/247
MA-ID-089	Gab-0932	−	−	−	11/04/11	M	162/166	233/245	175/179	274/310	231/231	314/314	215/227
MA-ID-090	Gab-0934	−	−	−	11/04/11	M	138/170	241/241	183/187	294/302	227/239	326/330	191/191
MA-ID-091	Gab-0939	−	−	−	11/04/11	F	162/166	225/241	179/183	298/306	231/259	318/322	191/191
MA-ID-092	Gab-0941	−	−	−	11/04/11	F	158/162	189/233	167/179	274/302	223/231	310/314	211/215
MA-ID-093	Gab-0948	−	−	−	14/04/11	M	146/150	201/225	171/175	294/298	227/235	310/322	239/243
	Gab-1466	−	/	/	09/03/12		146/150	201/225	171/175	294/298	227/235	310/322	239/243
	Gab-1469	−	/	/	09/03/12		146/150	201/225	171/175	294/298	227/235	310/322	239/243
	Gab-1470	−	/	/	09/03/12		146/150	201/225	171/175	294/298	227/235	310/322	239/243
MA-ID-094	Gab-0951	−	−	−	14/04/11	M	- /150	- /225	- /183	298/302	231/235	306/310	211/227
MAc-ID-095	Gab-0953	−	−	−	14/04/11	M	150/170	193/225	175/179	302/306	231/239	310/310	215/219
MA-ID-096	Gab-0954	−	−	−	14/04/11	M	146/150	225/225	171/179	302/306	231/235	310/310	211/243
	Gab-0961	−	−	−	14/04/11		146/150	225/225	171/179	302/306	231/235	310/310	211/243
MA-ID-097	Gab-0956	−	−	−	14/04/11	M	134/170	193/229	171/183	274/302	227/251	314/322	211/219
	Gab-1223	−	/	/	31/10/11		134/170	193/229	- /183	274/302	227/251	314/322	211/219
	Gab-1323	−	−	−	04/12/11		134/170	193/229	171/183	274/302	227/251	314/322	211/219

Table S1. *Cont.*

Individual MA site	Lab Code	Fecal Antibody Detection	RT-PCR		Sampling		Loci						
			pol	*gp41*	Date	Sex	D18S536	D4S243	D10S676	D9S922	D2S1326	D2S1333	D4S1627
MA-ID-098	Gab-2501	+	/	/	07/05/13	M	150/162	189/193	183/183	282/298	231/267	302/310	219/223
	Gab-2507	−	/	/	07/05/13		150/162	189/193	183/183	282/298	231/267	302/310	219/223
	Gab-2516	−	/	/	08/05/13		150/162	189/193	- /183	282/298	231/267	302/310	219/223
MA-ID-099	Gab-2504	−	/	/	07/05/13	M	162/170	189/189	179/183	294/298	235/243	314/322	- /211
	Gab-2520	+	/	/	08/05/13		162/170	- / -	179/183	294/298	235/243	314/322	211/211
MA-ID-100	Gab-0987	−	−	−	27/04/11	M	138/174	225/269	167/167	250/ -	231/251	306/306	211/223
MA-ID-101	Gab-0988	−	−	−	27/04/11	F	138/186	197/201	179/183	302/310	231/255	310/322	191/223
MA-ID-102	Gab-0990	−	−	−	27/04/11	F	138/162	189/201	167/167	298/298	227/231	326/334	- / -
MA-ID-103	Gab-0991	−	−	−	27/04/11	F	138/182	193/201	179/183	302/310	231/255	310/322	179/199
MA-ID-104	Gab-0994	−	−	−	04/05/11	M	162/166	225/229	175/179	298/298	- /227	306/322	223/227
	Gab-1335	−	/	−	01/12/11		162/166	225/229	175/179	298/298	227/227	306/322	223/227
MA-ID-105	Gab-0998	−	−	−	17/05/11	M	158/166	253/277	179/179	250/258	227/251	306/306	- / -
MA-ID-106	Gab-1002	−	−	−	17/05/11	M	166/170	225/245	179/187	294/302	227/251	306/322	191/223
MA-ID-107	Gab-1008	−	−	−	17/05/11	M	154/170	189/277	167/175	258/290	231/ -	306/318	- / -
MA-ID-108	Gab-2347	−	−	−	03/03/13	M	170/182	233/ -	183/ -	274/274	239/255	314/326	- / -
	Gab-2369	−	−	−	03/03/13		170/182	225/233	183/183	274/274	239/255	314/326	219/243
	Gab-2397	−	−	−	07/03/13		170/182	225/233	183/183	274/274	239/255	314/326	219/243
MA-ID-109	Gab-1022	−	−	−	20/05/11	M	166/170	189/277	179/183	298/302	223/231	310/314	211/211
MA-ID-110	Gab-1024	−	−	−	20/05/11	M	166/178	257/277	179/183	250/258	227/235	306/310	- / -
	Gab-1026	−	−	−	20/05/11		166/178	257/277	179/ -	250/258	227/235	306/310	211/ -
MA-ID-111	Gab-1468	−	/	/	09/03/12	F	138/170	225/245	183/191	290/302	231/231	306/310	219/ -
MA-ID-112	Gab-1028	−	−	−	07/06/11	F	150/170	209/241	175/179	294/302	223/255	310/314	219/231
	Gab-1030	−	−	−	07/06/11		150/170	209/241	175/179	294/302	223/255	310/314	219/231
MA-ID-113	Gab-1029	−	−	−	07/06/11	M	150/158	245/249	175/183	286/306	223/255	314/318	219/227
MA-ID-114	Gab-1032	−	−	−	07/06/11	M	162/166	197/225	175/187	298/318	231/247	306/306	191/223
MA-ID-115	Gab-1169	−	/	/	16/10/11	F	154/158	225/233	171/179	274/290	231/231	314/322	191/223

Table S1. *Cont.*

Individual MA site	Lab Code	Fecal Antibody Detection	RT-PCR		Sampling		Loci						
			pol	*gp41*	Date	Sex	D18S536	D4S243	D10S676	D9S922	D2S1326	D2S1333	D4S1627
	Gab-1170	−	/	/	16/10/11		154/158	225/233	171/179	274/290	231/251	314/322	191/223
Ma-ID-116	Gab-1464	−	/	/	09/03/12	F	134/138	225/225	179/187	286/302	243/251	314/314	211/223
MA-ID-117	Gab-1178	−	/	/	24/10/11	M	170/174	197/245	179/183	290/306	247/255	306/322	219/231
	Gab-1179	−	/	/	24/10/11		170/174	197/245	179/183	290/306	247/255	306/322	219/231
	Gab-1180	−	/	/	24/10/11		170/174	197/245	179/183	290/306	247/255	306/322	219/231
	Gab-1181	−	/	/	24/10/11		170/174	197/245	179/183	290/306	247/255	306/322	219/231
	Gab-1182	−	/	/	24/10/11		170/174	197/245	179/183	290/306	247/255	306/322	219/231
	Gab-1183	−	/	/	24/10/11		170/174	197/245	179/183	290/306	247/255	306/322	219/231
MA-ID-118	Gab-1261	−	/	/	06/11/11	M	154/170	221/225	171/179	290/294	231/235	306/310	219/223
Ma-ID-119	Gab-1508	−	/	/	11/03/12	F	154/166	225/229	167/167	290/298	235/247	314/322	231/239
	Gab-1514	−	/	/	11/03/12		154/166	225/229	167/ -	290/298	235/247	314/322	231/239
	Gab-1519	−	/	/	11/03/12		154/166	225/229	167/167	290/298	235/247	314/322	231/239
	Gab-1224	−	/	/	11/03/12		154/166	225/229	167/167	290/298	235/247	314/322	231/239
MA-ID-120	Gab-1225	−	/	/	02/11/11	M	146/166	225/245	167/179	290/314	223/231	310/330	191/223
MA-ID-121	Gab-1226	−	/	/	02/11/11	F	134/166	193/245	179/187	298/310	223/251	306/318	231/239
MA-ID-122	Gab-1236	−	/	/	02/11/11	F	166/170	201/225	147/179	302/306	231/243	306/322	223/243
MA-ID-123	Gab-1237	−	/	/	02/11/11	M	146/170	221/229	179/183	282/294	227/235	306/318	191/219
	Gab-1488	−	/	/	08/03/12		146/170	221/229	179/183	282/294	227/235	306/318	191/219
MA-ID-124	Gab-1243	−	/	/	06/11/11	M	162/170	193/225	179/187	294/298	223/231	306/306	211/215
	Gab-1244	−	/	/	06/11/11		162/170	193/225	179/187	294/298	223/231	306/306	211/215
	Gab-1245	−	/	/	06/11/11		162/170	193/225	179/ -	294/298	223/231	306/306	211/215
	Gab-1265	−	/	/	03/11/11		162/170	193/225	179/187	294/298	223/231	306/306	211/215
MA-ID-125	Gab-1246	−	/	/	06/11/11	F	146/154	193/229	179/187	286/290	239/255	322/330	211/243
	Gab-1258	−	/	/	06/11/11		146/154	193/229	179/187	286/290	239/255	322/330	211/243
MA-ID-126	Gab-1250	−	/	/	06/11/11	M	126/126	185/225	183/187	298/318	239/251	306/314	211/235
	Gab-1256	−	/	/	06/11/11		126/126	185/225	183/187	298/318	239/251	306/314	211/235

Table S1. *Cont.*

Individual MA site	Lab Code	Fecal Antibody Detection	RT-PCR		Sampling		Loci						
			pol	*gp41*	Date	Sex	D18S536	D4S243	D10S676	D9S922	D2S1326	D2S1333	D4S1627
	Gab-1260	−	/	/	06/11/11		126/-	- /225	183/187	298/318	239/251	306/314	211/235
	Gab-2291	−	/	/	19/02/13		126/-	- /225	183/187	298/318	239/251	306/314	211/235
	Gab-2295	−	/	/	19/02/13		126/126	185/225	183/187	298/318	239/251	306/314	211/235
	Gab-2309	−	−	+	04/03/13		126/126	185/225	183/187	298/318	239/251	306/314	211/235
	Gab-2310	−	−	−	04/03/13		126/126	185/225	183/187	298/318	239/251	306/314	211/235
MA-ID-127	Gab-2287	−	/	/	19/02/13	F	158/158	245/245	167/191	294/298	231/251	306/314	235/243
	Gab-2298	−	/	/	19/02/13		158/158	245/245	167/191	294/298	231/251	306/314	235/243
MA-ID-128	Gab-1262	−	/	/	03/11/11	M	150/166	225/225	175/183	302/302	223/247	306/306	227/235
MA-ID-129	Gab-2338	−	−	−	04/03/13	M	138/170	237/245	179/179	274/278	231/235	314/314	223/231
	Gab-2365	−	+	−	04/03/13		138/170	237/245	179/179	274/278	231/235	- /314	223/231
	Gab-2367	−	−	−	04/03/13		138/170	237/245	179/179	274/278	231/235	- /314	223/231
MA-ID-130	Gab-1277	−	/	/	01/12/11	M	134/150	205/225	179/179	290/302	231/235	310/318	235/239
	Gab-1278	−	/	/	01/12/11		134/150	205/225	179/179	290/302	231/235	310/318	235/239
MA-ID-131	Gab-1299	−	/	−	02/12/11	M	146/150	205/229	167/179	290/294	235/243	306/310	211/235
MA-ID-132	Gab-2357	−	−	−	03/03/13	M	138/166	197/225	175/187	298/302	235/243	306/314	227/239
	Gab-2400	−	−	−	07/03/13		138/166	197/225	175/187	298/302	235/243	306/314	227/239
MA-ID-133	Gab-2364	−	−	−	03/03/13	M	162/182	233/245	179/179	274/294	243/255	314/338	219/219
	Gab-2444	−	/	/	13/04/13		162/182	233/245	179/179	274/294	243/255	314/338	219/219
MA-ID-134	Gab-1305	−	/	/	02/12/11	M	134/162	193/225	167/191	282/290	223/255	302/306	231/239
MA-ID-135	Gab-1318	−	/	/	03/12/11	M	150/170	225/225	175/179	286/310	239/243	306/322	211/223
MA-ID-136	Gab-1326	−	/	/	05/12/11	M	150/150	201/233	175/187	286/302	227/231	310/318	231/235
MA-ID-137	Gab-1330	−	/	/	01/12/11	F	166/170	197/221	179/183	290/310	235/251	310/314	219/223
MA-ID-138	Gab-2373	−	−	−	06/03/13	F	146/154	221/225	167/183	290/306	227/235	306/322	211/227
	Gab-2399	−	−	−	07/03/13		146/154	221/225	167/183	290/306	227/235	306/322	211/227
MA-ID-139	Gab-1332	−	/	/	01/12/11	F	134/162	225/229	171/179	298/298	231/251	306/314	223/227
	Gab-2490	−	/	/	06/05/13		134/162	225/229	171/179	- /298	231/251	306/314	223/227

Table S1. *Cont.*

Individual MA site	Lab Code	Fecal Antibody Detection	RT-PCR		Sampling		Loci						
			pol	*gp41*	Date	Sex	D18S536	D4S243	D10S676	D9S922	D2S1326	D2S1333	D4S1627
MA-ID-140	Gab-1333	−	/	/	01/12/11	M	134/154	193/249	183/183	278/290	227/231	314/314	211/235
	Gab-2088	−	/	/	08/07/12		134/154	193/249	183/183	278/290	227/231	- /314	211/235
MA-ID-141	Gab-1334	−	/	/	01/12/11	F	170/174	225/225	183/183	290/290	235/247	302/310	219/227
	Gab-2527	−	/	/	09/05/13		170/174	225/225	183/183	290/290	235/247	302/310	219/227
	Gab-2529	−	/	/	09/05/13		170/174	225/225	183/183	290/290	235/247	302/310	219/227
MA-ID-142	Gab-2524	+	/	/	09/05/13	M	138/170	189/229	179/183	290/302	227/235	318/322	211/211
MA-ID-143	Gab-1340	−	/	/	01/12/11	F	162/174	197/229	175/183	290/294	223/227	314/322	211/243
MA-ID-144	Gab-1341	−	/	/	01/12/11	M	166/182	225/245	179/179	302/318	227/235	306/306	211/219
	Gab-1342	−	/	/	01/12/11		166/182	225/245	179/179	302/318	227/235	306/306	211/219
	Gab-2491	−	/	/	07/05/13		166/182	225/245	179/179	302/318	227/235	306/306	211/219
MA-ID-145	Gab-0901	−	−	−	07/04/11	F	162/166	201/225	167/175	298/302	227/255	306/310	223/223
	Gab-1343	−	/	/	01/12/11		162/166	201/225	167/175	298/302	227/255	306/310	223/223
	Gab-1964	−	/	/	26/10/12		- /166	201/225	167/175	298/302	227/255	306/310	223/ -
MA-ID-146	Gab-1344	−	/	/	01/12/11	F	154/158	185/197	175/183	286/306	227/231	306/318	227/231
MA-ID-147	Gab-1345	−	/	/	01/12/11	F	154/170	197/201	167/175	294/306	227/231	306/318	219/231
MA-ID-148	Gab-1346	−	/	/	01/12/11	M	146/154	197/225	179/183	298/306	231/235	- / 322	227/235
MA-ID-149	Gab-1347	−	/	/	03/12/11	M	146/170	229/229	183/183	294/306	223/235	318/322	211/235
MA-ID-150	Gab-2522	+	/	/	09/05/13	F	150/150	205/225	167/179	274/314	231/235	310/326	219/231
	Gab-2523	−	/	/	09/05/13		150/150	205/225	167/179	274/314	231/235	310/326	219/231
MA-ID-151	Gab-1365	−	/	/	03/12/11	M	170/170	197/221	167/175	290/306	235/251	306/310	191/227
	Gab-1366	−	/	/	03/12/11		170/170	197/221	167/175	290/306	235/251	306/310	191/227
	Gab-1368	−	/	/	08/12/11		170/170	197/221	167/175	290/306	235/251	306/310	191/227
	Gab-1370	−	/	/	08/12/11		170/170	197/221	167/175	290/306	235/251	306/310	191/227
	Gab-1372	−	/	/	08/12/11		170/170	197/221	167/175	290/306	235/251	306/310	191/227
MA-ID-152	Gab-2533	−	/	/	11/05/13	F	170/174	205/225	167/179	290/310	247/251	306/310	211/239
	Gab-2534	−	/	/	11/05/13		170/174	205/225	167/179	290/310	247/251	306/310	211/239

Table S1. *Cont.*

Individual MA site	Lab Code	Fecal Antibody Detection	RT-PCR		Sampling		Loci						
			pol	*gp41*	Date	Sex	D18S536	D4S243	D10S676	D9S922	D2S1326	D2S1333	D4S1627
	Gab-2610	−	/	/	30/05/13		170/174	205/225	167/179	290/310	247/251	306/310	211/239
MA-ID-153	Gab-1375	−	/	/	08/12/11	F	146/154	225/225	179/183	290/294	231/251	322/326	211/223
	Gab-1376	−	/	/	08/12/11		146/154	225/225	- / -	- / -	231/251	322/326	211/223
MA-ID-154	Gab-1477	−	/	/	10/03/12	F	134/146	201/225	179/183	298/298	235/243	310/318	219/235
	Gab-1478	−	/	/	10/03/12		134/146	201/225	179/183	298/298	235/243	310/318	219/235
	Gab-1479	−	/	/	10/03/12		134/146	201/225	179/183	298/298	235/243	310/318	219/235
MA-ID-155	Gab-1481	−	/	/	08/03/12	M	134/158	185/225	175/183	294/298	227/235	306/310	227/227
MA-ID-156	Gab-1465	−	/	/	09/03/12	F	154/158	225/225	175/195	302/306	239/247	306/314	- / -
	Gab-1494	−	/	/	08/03/12		154/158	225/225	175/195	302/306	239/247	306/314	191/243
MA-ID-157	Gab-1501	−	/	/	08/03/12	M	134/166	185/205	171/191	290/302	231/235	- / -	227/235
MA-ID-158	Gab-1505	−	/	/	11/03/12	F	166/174	201/241	183/183	274/290	235/247	318/322	219/227
MA-ID-159	Gab-1509	−	/	/	11/03/12	F	150/170	193/221	179/179	294/298	231/251	314/322	219/223
MA-ID-160	Gab-2514	−	/	/	08/05/13	M	150/170	221/221	179/183	274/290	235/243	314/314	219/227
MA-ID-161	Gab-1520	−	/	/	11/03/12	F	150/166	189/229	171/183	282/282	227/231	306/314	191/223
MA-ID-162	Gab-1521	−	/	/	11/03/12	F	158/170	225/225	167/187	298/302	231/235	310/322	223/239
MA-ID-163	Gab-1961	−	/	/	26/10/12	M	134/170	193/ -	167/183	270/302	231/251	310/318	231/ -
MA-ID-164	Gab-1962	+	−	−	26/10/12	F	166/174	193/209	179/187	282/314	215/231	322/ -	231/ -
MA-ID-165	Gab-2508	+	/	/	07/05/13	M	162/170	193/197	175/179	290/298	231/235	318/322	211/235
	Gab-2510	+	/	/	07/05/13		162/170	193/197	175/179	290/298	231/235	318/322	211/235
MA-ID-166	Gab-2500	+	/	/	07/05/13	F	150/170	193/221	179/183	298/298	- / -	310/314	219/235
MA-ID-167	Gab-1965	+	−	−	26/10/12	M	154/158	225/229	175/183	290/298	231/235	302/306	223/243
	Gab-2463	−	/	/	08/05/13		- / -	225/229	175/ -	- /298	231/235	302/306	223/243
MA-ID-168	Gab-1966	−	/	/	09/10/12	F	162/170	197/225	167/187	294/302	231/239	306/330	- / -
MA-ID-169	Gab-1970	+	−	−	10/10/12	M	146/170	185/245	179/179	290/302	231/247	314/326	231/239
MA-ID-170	Gab-1971	+	−	−	10/10/12	M	166/182	241/241	179/179	290/298	239/255	314/332	231/255
MA-ID-171	Gab-2499	−	/	/	07/05/13	M	162/170	217/229	183/183	298/298	- /235	302/330	215/219

Table S1. *Cont.*

Individual MA site	Lab Code	Fecal Antibody Detection	RT-PCR		Sampling		Loci						
			pol	*gp41*	Date	Sex	D18S536	D4S243	D10S676	D9S922	D2S1326	D2S1333	D4S1627
	Gab-2505	+	/	/	07/05/13		162/170	217/229	183/183	298/298	231/235	302/330	215/219
	Gab-2506	+	/	/	07/05/13		162/170	217/229	183/183	298/298	- /235	302/330	215/219
	Gab-2511	+	/	/	08/05/13		162/170	217/229	183/183	298/298	231/235	302/330	215/219
	Gab-2515	+	/	/	08/05/13		162/170	217/229	183/183	298/298	- /235	302/330	215/219
	Gab-2517	+	/	/	08/05/13		162/170	217/229	183/183	298/298	231/235	302/330	215/219
	Gab-2519	−	/	/	08/05/13		162/170	217/229	183/183	298/298	231/235	302/330	215/219
MA-ID-172	Gab-1973	+	−	−	10/11/12	M	134/158	229/245	179/179	298/302	231/235	306/338	215/227
MA-ID-173	Gab-1974	−	/	/	10/11/12	M	134/138	221/245	179/179	290/302	227/227	306/314	191/215
MA-ID-174	Gab-2495	−	/	/	07/05/13	M	- /170	193/229	167/179	290/294	235/243	314/322	227/231
	Gab-2496	−	/	/	07/05/13		- /170	193/229	167/179	290/294	235/243	314/322	227/231
	Gab-2497	−	/	/	07/05/13		- /170	193/229	167/179	- /294	235/243	314/322	227/231
	Gab-2498	−	/	/	07/05/13		134/170	193/229	167/179	290/294	235/243	314/322	227/231
MA-ID-175	Gab-2085	+	−	−	06/07/12	F	142/174	225/225	171/183	294/302	231/235	318/322	207/231
MA-ID-176	Gab-2087	+	−	−	08/12/12	F	150/166	201/201	183/183	298/306	243/243	306/318	215/235
Ma-ID-177	Gab-2494	+	/	/	07/05/13	M	162/170	197/245	179/183	298/298	235/235	302/310	219/219
MA-ID-178	Gab-2090	−	/	/	08/12/12	M	166/170	193/225	179/183	298/302	223/231	310/314	211/223
	Gab-2091	−	/	/	08/12/12		166/170	193/225	179/183	298/302	223/231	310/314	211/223
MA-ID-179	Gab-2458	−	/	/	08/05/13	M	126/170	193/221	179/183	290/302	231/235	310/318	223/235
	Gab-2474	−	/	/	08/05/13		126/170	193/221	179/183	290/302	231/235	310/318	223/235
	Gab-2477	−	/	/	08/05/13		126/170	193/221	179/183	290/302	231/235	310/318	223/235
MA-ID-180	Gab-2493	+	/	/	07/05/13	M	146/170	221/229	167/175	274/290	231/235	314/322	211/227
	Gab-2503	−	/	/	07/05/13		146/170	221/229	167/175	274/290	231/235	314/322	211/227
	Gab-2509	−	/	/	08/05/13		146/170	221/229	167/175	274/290	231/235	314/322	211/227
	Gab-2518	−	/	/	08/05/13		146/170	221/229	167/175	274/290	231/235	314/322	211/227
MA-ID-181	Gab-2093	+	−	−	12/12/12	F	146/178	229/241	183/183	298/314	227/231	306/314	239/243
MA-ID-182	Gab-2094	−	/	/	12/12/12	F	158/162	- / -	179/183	270/286	227/255	310/322	179/239

Table S1. *Cont.*

Individual MA site	Lab Code	Fecal Antibody Detection	RT-PCR		Sampling		Loci						
			pol	*gp41*	Date	Sex	D18S536	D4S243	D10S676	D9S922	D2S1326	D2S1333	D4S1627
MA-ID-183	Gab-2492	+	/	/	02/05/13	M	150/170	217/233	179/179	286/298	231/231	306/314	211/243
MA-ID-184	Gab-2098	−	/	/	12/12/12	F	150/174	201/229	179/183	290/294	235/243	306/322	227/243
MA-ID-185	Gab-2100	+	+	−	07/12/12	M	150/170	193/229	167/183	290/294	231/235	302/322	211/231
	Gab-2512	+	/	/	08/05/13		150/170	193/229	- / -	290/294	231/235	302/322	211/231
MA-ID-186	Gab-2269	−	/	/	19/02/13	F	134/170	193/245	175/179	298/298	231/235	310/322	219/227
	Gab-2285	−	/	/	19/02/13		134/170	193/245	175/179	298/298	231/235	310/322	219/227
MA-ID-187	Gab-2268	−	/	/	07/02/13	M	150/166	225/237	167/171	302/306	235/243	314/322	191/235
MA-ID-188	Gab-2270	+	/	/	07/02/13	F	150/154	201/221	171/179	290/318	231/251	318/322	223/231
	Gab-2304	+	/	/	19/02/13		- /154	201/221	171/179	290/318	231/251	318/322	223/231
MA-ID-189	Gab-2271	+	/	/	19/02/13	M	138/162	201/229	167/167	298/302	235/235	326/334	215/223
	Gab-2275	+	/	/	19/02/13		138/162	201/229	167/167	298/302	235/235	326/334	215/223
	Gab-2323	+	−	−	04/03/13		138/162	201/229	167/167	298/302	235/235	326/ -	215/223
	Gab-2326	+	−	−	04/03/13		138/162	201/229	167/167	298/302	235/235	326/334	215/223
	Gab-2327	+	−	−	04/03/13		138/162	201/229	167/167	298/302	235/235	326/334	215/223
	Gab-2329	+	−	−	04/03/13		138/162	201/229	167/167	298/302	235/235	326/334	215/223
	Gab-2331	+	−	−	04/03/13		138/162	201/229	167/167	298/302	235/235	326/334	215/223
MA-ID-190	Gab-2274	−	/	/	19/02/13	F	146/146	221/229	179/179	286/290	239/251	306/330	207/243
MA-ID-191	Gab-2277	+	/	/	19/02/13	M	126/154	225/229	167/183	290/318	227/251	306/314	211/235
	Gab-2278	+	/	/	19/02/13		126/154	225/229	167/183	290/318	227/251	306/314	211/235
MA-ID-192	Gab-2483	−	/	/	06/05/13	F	150/170	221/225	187/195	294	231/235	302/306	223/227
MA-ID-193	Gab-2283	−	/	/	19/02/13	F	146/166	201/225	167/175	302/306	227/231	310/310	215/231
	Gab-2328	−	−	−	04/03/13		146/166	201/225	167/175	302/306	227/231	310/310	215/231
	Gab-2332	−	−	−	04/03/13		146/166	201/225	167/175	302/306	227/231	310/310	215/231
MA-ID-194	Gab-2284	−	/	/	19/02/13	F	158/158	245/ -	167/175	- / -	215/231	306/314	243/235
MA-ID-195	Gab-2286	−	/	/	19/02/13	F	146/166	205/225	183/183	282/302	235/259	310/314	215/219
	Gab-2318	−	−	−	04/03/13		146/166	205/225	183/183	282/302	235/259	310/314	215/219

Table S1. *Cont.*

Individual MA site	Lab Code	Fecal Antibody Detection	RT-PCR		Sampling		Loci						
			pol	*gp41*	Date	Sex	D18S536	D4S243	D10S676	D9S922	D2S1326	D2S1333	D4S1627
	Gab-2319	−	−	−	04/03/13		146/166	205/225	- /183	282/ -	235/259	310/314	215/219
MA-ID-196	Gab-2481	−	/	/	06/05/13	F	138/154	225/249	183/183	290/294	227/255	314/322	235/239
	Gab-2482	−	/	/	06/05/13		138/154	225/249	183/183	290/294	227/255	314/322	235/239
MA-ID-197	Gab-2480	−	/	/	06/05/13	F	162/170	201/229	175/179	302/314	231/231	306/330	231/235
MA-ID-198	Gab-2289	−	/	/	19/02/13	F	126/146	221/229	179/183	290/302	235/251	- / -	- / -
MA-ID-199	Gab-2294	+	/	/	19/02/13	F	154/158	185/225	175/179	302/314	231/239	306/310	215/219
	Gab-2305	−	−	−	03/03/13		154/158	185/225	175/179	302/314	231/239	306/310	215/219
	Gab-2334	+	−	−	04/03/13		154/158	185/225	175/179	302/314	231/239	306/310	215/219
MA-ID-200	Gab-2475	−	/	/	08/05/13	M	162/170	225/241	175/183	294/298	227/247	322/330	215/223
MA-ID-201	Gab-2472	−	/	/	08/05/13	M	142/174	229/229	167/167	- / -	235/255	302/322	223/227
MA-ID-202	Gab-2456	+	/	/	08/05/13	F	134/170	241/245	183/183	290/294	235/247	302/330	215/231
MA-ID-203	Gab-2306	−	−	+	04/03/13	M	154/154	181/201	167/183	290/298	223/247	306/326	223/235
MA-ID-204	Gab-2311	+	−	−	04/03/13	M	158/182	209/225	175/179	290/294	223/251	322/322	211/219
	Gab-2312	+	−	−	04/03/13		158/182	209/225	175/179	290/294	223/251	322/322	211/219
	Gab-2313	+	−	+	04/03/13		158/182	209/225	175/179	290/294	223/251	322/322	211/219
	Gab-2314	+	−	−	04/03/13		158/182	209/225	175/179	290/294	223/251	322/322	211/219
	Gab-2315	+	−	−	04/03/13		158/182	209/225	175/179	290/294	223/251	322/322	211/219
	Gab-2316	+	−	+	04/03/13		158/182	209/225	175/179	290/294	223/251	- / -	211/219
MA-ID-205	Gab-2317	−	−	−	04/03/13	M	162/162	201/205	167/183	298/298	235/239	- / -	- / -
	Gab-2330	−	−	−	04/03/13		- /162	201/205	167/183	298/298	235/239	306/322	215/223
	Gab-2333	−	−	−	04/03/13		162/162	201/205	167/183	298/298	235/239	306/322	215/223
MA-ID-206	Gab-2470	−	/	/	08/05/13	F	170/170	193/225	167/183	290/294	231/231	306/310	219/235
MA-ID-207	Gab-2320	+	−	−	04/03/13	F	170/170	205/225	179/183	294/306	247/259	306/322	223/227
	Gab-2321	+	−	−	04/03/13		170/170	205/225	179/183	294/306	247/259	306/322	223/227
MA-ID-208	Gab-2361	−	−	−	03/03/13	M	138/170	225/237	179/183	274/286	231/235	306/314	207/223
	Gab-2389	−	−	−	07/03/13		138/170	225/237	179/183	274/286	231/235	306/314	207/223

Table S1. *Cont.*

Individual MA site	Lab Code	Fecal Antibody Detection	RT-PCR *pol*	RT-PCR *gp41*	Sampling Date	Sampling Sex	Loci D18S536	D4S243	D10S676	D9S922	D2S1326	D2S1333	D4S1627
MA-ID-209	Gab-2469	−	/	/	08/05/13	M	134/170	193/249	167/183	302/306	231/251	310/318	231/231
	Gab-2471	−	/	/	08/05/13		134/170	193/249	167/183	302/306	231/251	310/318	- /231
	Gab-2476	+	/	/	08/05/13		134/170	193/249	167/183	302/306	231/251	310/318	231/231
MA-ID-210	Gab-2335	−	+	+	03/03/13	F	138/166	185/197	175/175	298/302	- /235	306/314	231/239
	Gab-2339	−	+	+	03/03/13		138/166	185/197	175/175	298/302	235/235	306/314	231/239
	Gab-2392	−	−	−	07/03/13		138/166	185/197	175/175	298/302	235/235	306/314	231/239
MA-ID-211	Gab-2336	−	−	−	04/03/13	F	138/154	225/237	179/183	278/290	227/235	314/322	211/223
MA-ID-212	Gab-2337	−	−	−	04/03/13	M	170/182	197/245	167/179	274/298	223/231	306/322	223/239
	Gab-2343	−	−	−	03/03/13		170/182	197/245	167/179	274/298	223/231	306/322	223/239
	Gab-2379	−	−	−	06/03/13		170/182	197/245	167/179	274/298	223/231	306/322	223/239
MA-ID-213	Gab-2445	−	/	/	15/04/13	F	134/150	185/225	179/183	274/306	227/247	314/326	215/219
	Gab-2447	−	/	/	15/04/13		134/150	185/225	179/183	274/306	227/247	314/326	215/219
	Gab-2448	−	/	/	15/04/13		134/150	185/225	179/183	274/306	227/247	314/326	215/219
	Gab-2449	−	/	/	15/04/13		134/150	185/225	179/183	274/306	227/247	314/326	215/219
	Gab-2450	−	/	/	15/04/13		134/150	185/225	179/183	274/306	227/247	314/326	215/219
	Gab-2451	−	/	/	15/04/13		134/150	185/225	179/183	274/306	227/247	314/326	215/219
	Gab-2452	−	/	/	15/04/13		134/150	185/225	179/183	274/306	227/247	314/326	215/219
	Gab-2454	−	/	/	15/04/13		134/150	185/225	179/183	274/306	227/247	314/326	215/219
	Gab-2455	−	/	/	15/04/13		134/150	185/225	179/183	274/306	227/247	314/326	215/219
MA-ID-214	Gab-2393	+	−	−	07/03/13	M	138/158	225/245	179/183	278/306	223/235	302/306	223/239
MA-ID-215	Gab-2340	−	−	−	03/03/13	M	166/170	185/237	179/183	302/302	235/235	302/322	223/223
	Gab-2348	−	−	−	03/03/13		166/170	185/237	179/183	302/302	235/235	302/322	223/223
	Gab-2359	−	−	−	03/03/13		166/170	185/237	179/183	302/302	235/235	302/322	223/223
	Gab-2362	−	−	−	03/03/13		166/170	185/237	179/183	302/302	235/235	302/322	223/223
	Gab-2363	−	−	−	03/03/13		166/170	185/237	179/183	302/302	235/235	302/322	223/223
	Gab-2378	−	−	−	06/03/13		166/170	185/237	179/183	302/302	- /235	302/322	223/223

Table S1. *Cont.*

Individual MA site	Lab Code	Fecal Antibody Detection	RT-PCR		Sampling		Loci						
			pol	*gp41*	Date	Sex	D18S536	D4S243	D10S676	D9S922	D2S1326	D2S1333	D4S1627
	Gab-2388	−	−	−	06/03/13		166/170	185/237	179/183	302/ -	235/235	302/322	223/ -
	Gab-2391	−	−	−	07/03/13		166/170	185/237	179/183	302/302	235/235	302/322	223/223
	Gab-2387	−	−	−	06/03/13		166/170	185/237	179/183	- /302	235/235	302/322	223/ -
MA-ID-216	Gab-2342	−	−	−	03/03/13	M	134/138	225/ -	175/179	- / -	227/243	306/322	227/231
	Gab-2382	−	−	−	06/03/13		134/138	225/225	175/179	298/302	227/243	306/322	227/231
	Gab-2383	−	−	−	06/03/13		134/138	225/225	175/179	298/302	227/243	306/322	227/231
	Gab-2402	−	−	−	07/03/13		134/138	225/225	175/179	298/302	227/243	306/322	227/231
MA-ID-217	Gab-2385	−	−	−	06/03/13	M	150	221/233	167/179	294/294	231/243	310/338	227/227
MA-ID-218	Gab-2345	−	−	+	03/03/13	M	166/182	185/245	175/179	298/306	231/235	306/326	223/239
	Gab-2351	−	+	−	03/03/13		166/182	185/245	175/179	298/306	231/235	306/326	223/239
	Gab-2353	−	−	+	03/03/13		166/182	185/245	175/179	298/306	231/235	306/326	223/ -
	Gab-2354	−	−	−	03/03/13		166/182	185/245	175/179	298/306	231/235	306/326	223/239
	Gab-2355	−	+	+	03/03/13		166/182	185/245	175/179	298/306	231/235	306/326	223/239
	Gab-2356	−	−	−	03/03/13		166/182	185/245	175/179	298/306	231/235	306/326	223/239
	Gab-2358	−	−	+	03/03/13		166/182	185/245	175/179	298/306	231/235	306/326	223/239
	Gab-2360	−	−	+	03/03/13		166/182	185/245	175/179	298/306	231/235	306/326	223/239
	Gab-2374	−	−	−	06/03/13		166/182	185/245	175/179	298/306	231/235	306/326	223/239
	Gab-2376	−	−	−	06/03/13		166/182	185/245	175/179	298/306	231/235	306/326	223/239
	Gab-2395	−	−	−	07/03/13		166/182	185/245	175/179	298/306	231/235	306/326	223/239
	Gab-2396	−	−	−	07/03/13		166/182	185/245	175/179	298/306	231/235	306/326	223/239
	Gab-2398	−	−	−	07/03/13		166/182	185/245	175/179	298/306	231/235	306/326	223/239
MA-ID-219	Gab-2346	−	−	−	03/03/13	M	150/170	185/241	171/179	302/306	243/263	306/314	215/223
	Gab-2352	−	−	−	03/03/13		150/170	185/241	171/179	302/306	243/263	306/314	215/223
	Gab-2372	−	−	−	06/03/13		150/170	185/241	171/179	302/306	243/263	306/314	215/223
	Gab-2377	−	−	−	06/03/13		150/170	185/241	171/179	302/306	243/263	306/314	215/223
MA-ID-220	Gab-2381	−	−	−	06/03/13	F	138/146	229/245	167/179	294/294	223/247	314/322	231/235

Table S1. *Cont.*

Individual MA site	Lab Code	Fecal Antibody Detection	RT-PCR		Sampling		Loci						
			pol	*gp41*	Date	Sex	D18S536	D4S243	D10S676	D9S922	D2S1326	D2S1333	D4S1627
MA-ID-221	Gab-2344	−	−	−	03/03/13	M	138/146	221/225	167/187	290/294	235/243	322/338	211/219
	Gab-2349	−	−	−	03/03/13		138/146	221/225	167/187	290/294	235/243	322/338	211/219
	Gab-2370	−	−	−	03/03/13		138/146	221/225	167/187	290/294	235/243	322/338	211/219
	Gab-2375	−	−	−	06/03/13		138/146	221/225	167/187	290/294	235/243	322/338	211/219
	Gab-2386	−	−	−	06/03/13		138/146	221/225	167/187	290/294	235/243	322/338	211/219
	Gab-2394	−	−	−	07/03/13		138/146	221/225	167/187	290/294	235/243	322/338	211/219
	Gab-2401	−	−	−	07/03/13		138/146	221/225	167/187	290/294	235/243	322/338	211/219
	Gab-2403	−	−	−	07/03/13		138/146	221/225	167/187	290/294	235/243	322/338	211/219
	Gab-2404	+	−	−	07/03/13		138/146	- / -	167/187	290/294	235/243	- / -	211/219
MA-ID-222	Gab-2350	−	−	−	03/03/13	M	170/170	217/225	179/183	250/298	231/235	310/322	215/239
MA-ID-223	Gab-2265	−	/	/	20/12/12	F	170/170	185/233	151/179	274/298	- / -	322/322	- / -
MA-ID-224	Gab-2380	−	−	−	06/03/13	F	138/146	225/229	175/179	294/302	223/235	314/322	227/231
	Gab-2384	−	−	−	06/03/13		138/146	225/229	175/179	294/302	223/235	314/322	227/231
**ML site**													
ML-ID-01	Gab-1406	+	+	−	16/01/12	F	162/170	225/229	167/179	290/306	231/235	322/326	223/227
ML-ID-02	Gab-1120	+	+	−	21/07/11	M	150/170	221/237	167/183	302/302	235/255	306/310	231/231
ML-ID-03	Gab-1387	+	+	−	16/01/12	F	134/170	225/237	179/187	282/306	247/255	306/306	211/227
	Gab-1394	+	+	−	16/01/12		134/170	225/237	179/187	282/306	247/255	306/306	211/227
	Gab-1398	+	−	−	16/01/12		134/170	225/237	179/187	282/306	247/255	306/306	211/227
	Gab-1399	+	−	−	16/01/12		134/170	225/237	179/187	282/306	247/255	306/306	211/227
	Gab-1402	+	−	−	16/01/12		134/170	225/237	179/187	282/306	247/255	306/306	211/227
	Gab-1403	+	−	−	16/01/12		134/170	225/237	179/187	282/306	247/255	306/306	211/227
	Gab-1407	+	−	−	16/01/12		134/170	225/237	179/187	282/306	247/255	306/306	211/227
	Gab-1427	+	+	+	16/01/12		134/170	225/237	179/187	282/306	247/255	306/306	211/227
	Gab-1428	+	−	−	16/01/12		134/170	225/237	179/187	282/306	247/255	306/306	211/227
	Gab-1429	+	+	−	16/01/12		134/170	225/237	179/187	282/306	247/255	306/306	211/227

Table S1. *Cont.*

Individual MA site	Lab Code	Fecal Antibody Detection	RT-PCR		Sampling		Loci						
			pol	*gp41*	Date	Sex	D18S536	D4S243	D10S676	D9S922	D2S1326	D2S1333	D4S1627
	Gab-1430	+	−	−	16/01/12		134/170	225/237	179/187	282/306	247/255	306/306	211/227
	Gab-1431	+	−	−	16/01/12		134/170	225/237	179/187	282/306	247/255	306/306	211/227
	Gab-1435	+	−	−	16/01/12		134/170	225/237	179/187	282/306	247/255	306/306	211/227
	Gab-1440	+	−	−	16/01/12		134/170	225/237	179/187	282/306	247/255	306/306	211/227
ML-ID-04	Gab-1389	+	−	−	16/01/12	F	162/170	225/229	167/179	290/306	231/235	302/322	223/227
	Gab-1391	+	−	−	16/01/12		162/170	225/229	167/179	290/306	231/235	302/322	223/227
	Gab-1393	+	−	−	16/01/12		162/170	225/229	167/179	290/306	231/235	302/322	223/227
	Gab-1395	+	−	−	16/01/12		162/170	225/229	167/179	290/306	231/235	302/322	223/227
	Gab-1396	+	−	−	16/01/12		162/170	225/229	167/179	290/306	231/235	302/322	223/227
	Gab-1401	+	−	−	16/01/12		162/170	225/229	167/179	290/306	231/235	302/322	223/227
	Gab-1404	+	−	−	16/01/12		162/170	225/229	167/179	290/306	231/235	302/322	223/227
	Gab-1408	+	−	−	16/01/12		162/170	225/229	167/179	290/306	231/235	302/322	223/227
	Gab-1432	+	+	−	16/01/12		162/170	225/229	167/179	290/306	231/235	302/322	223/227
	Gab-1436	+	−	−	16/01/12		162/170	225/ -	167/179	290/306	231/ -	302/322	223/227
	Gab-1437	+	−	−	16/01/12		162/170	225/229	167/179	290/306	231/235	302/322	223/227
	Gab-1438	+	−	−	16/01/12		162/170	225/229	167/179	290/306	231/235	302/322	223/227
	Gab-1439	+	−	−	16/01/12		162/170	225/229	167/179	290/306	231/235	302/322	223/227
ML-ID-05	Gab-1379	−	/	/	11/01/12	F	158/166	197/209	167/175	290/306	223/239	310/322	227/231
	Gab-1380	−	/	/	11/01/12		158/166	197/209	167/175	290/306	223/239	310/322	227/231
ML-ID-06	Gab-1390	+	−	−	16/01/12	F	134/150	201/237	179/187	282/298	255/263	306/306	227/239
ML-ID-07	Gab-1422	+	−	−	11/01/12	F	154/166	181/181	183/191	254/262	231/255	290/294	187/191
ML-ID-08	Gab-1397	+	−	−	16/01/12	F	162/170	-/257	167/179	290/306	231/235	302/322	223/227
	Gab-1400	+	−	−	16/01/12		162/170	229/257	167/179	290/306	231/235	302/322	223/227
ML-ID-09	Gab-1818	−	/	/	13/09/12	M	162/170	225/237	175/183	274/294	231/235	306/322	215/227
	Gab-1819	−	/	/	13/09/12		162/170	225/237	175/183	274/294	231/235	306/322	215/227
	Gab-1820	−	/	/	13/09/12		162/170	225/237	175/183	274/294	231/235	306/322	215/227

Table S1. *Cont.*

Individual MA site	Lab Code	Fecal Antibody Detection	RT-PCR		Sampling		Loci						
			pol	*gp41*	Date	Sex	D18S536	D4S243	D10S676	D9S922	D2S1326	D2S1333	D4S1627
	Gab-1822	−	/	/	13/09/12		162/170	225/237	175/183	274/294	231/235	306/322	215/227
	Gab-1825	−	/	/	13/09/12		162/ -	225/237	175/183	274/294	231/235	306/322	215/227
	Gab-1827	−	/	/	13/09/12		162/170	225/237	175/183	274/294	231/235	306/322	215/227
	Gab-1828	−	/	/	13/09/12		162/170	225/237	175/183	274/294	231/235	306/322	215/227
	Gab-1829	−	/	/	13/09/12		162/170	225/237	175/183	274/294	231/235	306/322	215/227
	Gab-1830	−	/	/	13/09/12		162/170	225/237	175/183	274/294	231/235	306/322	215/227
	Gab-1835	−	/	/	13/09/12		162/170	-/ -	175/183	274/294	231/235	306/ -	215/227
	Gab-1852	−	/	/	14/09/12		162/170	225/ -	175/183	274/-	231/235	-/ -	215/227
	Gab-1856	−	/	/	14/09/12		162/170	225/ -	175/183	274/ -	231/235	306/322	215/227
	Gab-1854	−	/	/	14/09/12		162/170	225/ -	175/183	274/ -	231/235	306/322	215/227
	Gab-1855	−	/	/	14/09/12		162/170	-/ -	175/183	-/ -	231/235	306/322	215/227
ML-ID-10	Gab-1842	−	/	/	14/09/12	M	134/150	197/ -	167/167	-/302	255/255	310/318	231/279
	Gab-1843	+	/	/	14/09/12		134/150	197/ -	167/167	274/302	255/255	310/318	231/279
	Gab-1846	−	/	/	14/09/12		134/150	197/ -	167/ -	274/302	255/255	310/318	231/279
	Gab-1882	−	/	/	14/09/12		134/150	197/237	167/167	274/302	255/255	310/318	231/231
	Gab-1883	−	/	/	14/09/12		134/150	197/237	167/167	274/302	255/255	310/318	231/231
	Gab-1884	−	/	/	14/09/12		134/150	197/237	167/167	274/302	255/255	310/318	231/231
	Gab-1895	−	/	/	15/09/12		134/150	197/237	167/167	274/302	255/255	310/318	231/231
	Gab-1899	−	/	/	15/09/12		134/150	197/237	167/167	274/302	-/255	310/318	231/231
ML-ID-11	Gab-1918	+	−	−	14/09/12	F	154/158	177/237	179/187	258/ -	215/247	318/326	231/231
ML-ID-12	Gab-1844	+	−	−	13/09/12	M	134/158	173/197	167/183	274/302	255/267	310/318	231/279
ML-ID-13	Gab-1858	+	+	−	14/09/12	F	158/170	-/221	179/183	286/302	-/267	318/326	219/239
ML-ID-14	Gab-1868	−	/	/	14/09/12	F	150/170	229/241	167/187	298/302	231/243	302/306	219/239
ML-ID-15	Gab-1869	−	/	/	14/09/12	M	166/170	193/197	175/175	282/290	231/239	306/322	231/235
	Gab-1891	−	/	/	14/09/12		166/170	193/197	175/175	282/290	231/239	306/322	231/235
ML-ID-16	Gab-1892	−	/	/	15/09/12	F	134/150	193/229	179/183	290/298	235/243	310/322	219/239

Table S1. *Cont.*

Individual MA site	Lab Code	Fecal Antibody Detection	RT-PCR *pol*	RT-PCR *gp41*	Sampling Date	Sex	Loci D18S536	D4S243	D10S676	D9S922	D2S1326	D2S1333	D4S1627
	Gab-1872	−	/	/	14/09/12		134/150	193/229	179/183	290/298	235/243	310/322	219/239
	Gab-1873	−	/	/	14/09/12		134/150	193/229	179/183	290/298	235/243	310/322	219/239
	Gab-1874	−	/	/	14/09/12		134/150	193/229	179/183	290/298	235/243	310/322	219/239
	Gab-1875	+	−	−	14/09/12		134/150	193/229	179/183	290/298	235/243	310/322	219/239
	Gab-1876	−	/	/	14/09/12		134/150	193/229	179/183	290/298	235/243	310/322	219/239
	Gab-1878	−	/	/	14/09/12		134/150	193/229	179/183	290/298	235/243	310/322	219/239
	Gab-1879	−	/	/	14/09/12		134/150	193/229	179/183	290/298	235/243	310/322	219/239
	Gab-1880	−	/	/	14/09/12		134/150	193/229	179/183	290/298	235/243	310/322	219/239
	Gab-1881	−	/	/	14/09/12		134/150	193/229	179/183	290/298	235/243	310/322	219/239
ML-ID-17	Gab-1885	−	/	/	14/09/12	F	158/158	193/217	175/183	298/302	231/231	318/322	219/243
	Gab-1886	−	/	/	14/09/12		158/158	193/217	175/183	298/302	231/231	318/322	219/243
	Gab-1887	−	/	/	14/09/12		158/158	193/217	175/183	298/302	231/231	318/322	219/243
ML-ID-18	Gab-1888	−	/	/	14/09/12	M	150/170	225/241	175/ -	294/ -	231/251	306/310	227/231
ML-ID-19	Gab-1894	−	/	/	15/09/12	M	166/174	193/197	175/175	282/290	231/239	306/322	-/ -
**IY site**													
IY-ID-01	Gab-2103	−	/	/	06/12/12	M	134/150	201/ -	183/191	290/298	215/227	318/322	211/ -
IY-ID-02	Gab-2102	−	/	/	06/12/12	M	134/150	225/ -	183/191	290/290	187/215	-/ -	211/ -
IY-ID-03	Gab-2106	−	/	/	06/12/12	F	170/174	225/245	183/183	274/286	215/235	302/ -	223/223
IY-ID-04	Gab-2108	+	−	−	07/12/12	M	150/166	201/241	179/183	290/302	243/255	306/318	211/231
	Gab-2109	+	−	−	07/12/12		150/166	201/241	179/183	290/302	243/255	306/318	211/231
	Gab-2110	+	−	−	07/12/12		150/166	201/241	179/183	290/302	243/255	306/318	211/231
	Gab-2111	+	−	−	07/12/12		150/166	201/241	179/183	290/302	243/255	306/318	211/231
	Gab-2112	+	−	−	07/12/12		150/166	201/241	179/183	290/302	243/255	306/318	211/231
	Gab-2113	+	−	−	07/12/12		150/166	201/241	179/183	290/302	243/255	306/318	211/231
	Gab-2114	+	−	−	07/12/12		150/166	201/241	179/183	290/302	243/255	306/318	211/231
	Gab-2115	+	−	−	07/12/12		150/166	201/241	179/183	290/302	243/255	306/318	211/231

Table S1. *Cont.*

Individual MA site	Lab Code	Fecal Antibody Detection	RT-PCR		Sampling		Loci						
			pol	*gp41*	Date	Sex	D18S536	D4S243	D10S676	D9S922	D2S1326	D2S1333	D4S1627
	Gab-2116	+	+	−	07/12/12		150/166	201/241	179/183	290/302	243/255	306/318	211/231
	Gab-2117	+	−	−	07/12/12		150/166	201/241	179/183	290/302	243/255	306/318	211/231
IY-ID-05	Gab-2120	−	/	/	10/12/12	F	146/186	225/245	179/183	278/306	235/243	306/314	227/235
IY-ID-06	Gab-2136	−	/		07/12/12	F	138/170	221/229	183/199	294/306	223/227	306/318	-/243
	Gab-2141	−	/	/	07/12/12		138/170	221/229	183/199	294/306	223/227	306/318	223/243
IY-ID-07	Gab-2137	+	−	−	07/12/12	F	154/166	225/225	179/187	302/306	235/239	306/310	227/243
IY-ID-08	Gab-2140	−	/	/	07/12/12	M	138/166	185/225	179/179	290/302	239/255	306/322	219/243
IY-ID-09	Gab-2142	+	−	−	09/12/12	M	150182	225/225	179/183	298/310	255/255	318/330	219/231
	Gab-2146	+	−	−	09/12/12		150/150	-/225	179/183	298/310	255/255	318/330	219/231
IY-ID-10	Gab-2143	+	−	−	09/12/12	F	146/170	197/221	179/183	298/302	231/235	302/314	219/235
	Gab-2144	+	−	−	09/12/12		146/170	197/221	179/183	298/302	231/235	302/314	219/235
	Gab-2145	+	−	−	09/12/12		146/170	197/221	179/183	298/302	231/235	302/314	219/235
IY-ID-11	Gab-2149	−	/	/	09/12/12	M	150/170	217/225	179/183	274/298	231/231	306/310	219/227
IY-ID-12	Gab-2148	−	/	/	09/12/12	M	-/150	217/225	179/179	274/298	231/255	306/310	219/235
**OD site**													
OD-ID-01	Gab-2405	−	/	/	04/04/13	M	158/166	245/245	167/183	298/306	231/251	310/330	219/239
	Gab-2406	−	/	/	04/04/13		158/166	245/245	167/183	298/306	231/251	310/330	219/239
	Gab-2407	−	/	/	04/04/13		158/166	245/245	167/183	298/306	231/251	310/330	219/239
OD-ID-02	Gab-2408	−	/	/	04/04/13	F	150/174	193/225	175/179	290/314	243/247	306/310	219/235
	Gab-2409	−	/	/	04/04/13		150/174	193/ -	-/ -	290/314	243/247	310/310	219/235
OD-ID-03	Gab-2443	−	/	/	11/04/13	M	134/162	201/249	175/179	302/306	231/231	318/322	191/219
OD-ID-04	Gab-2416	+	−	−	04/04/13	M	158/170	205/225	167/183	254/302	235/255	322/ -	219/231
	Gab-2419	+	−	−	04/04/13		158/170	205/225	167/183	302/302	235/255	306/322	219/231
OD-ID-05	Gab-2412	−	/	/	04/04/13	F	174/174	-/ -	175/179	290/314	215/243	310/ -	235/ -
OD-ID-06	Gab-2422	−	/	/	04/04/13	M	170/174	237/237	179/179	302/302	235/235	306/322	219/231
	Gab-2423	−	/	/	08/04/13		170/174	237/237	179/179	302/302	235/235	306/322	219/231

Table S1. *Cont.*

Individual MA site	Lab Code	Fecal Antibody Detection	RT-PCR		Sampling		Loci						
			pol	*gp41*	Date	Sex	D18S536	D4S243	D10S676	D9S922	D2S1326	D2S1333	D4S1627
	Gab-2424	−	/	/	08/04/13		170/174	237/237	179/179	302/302	235/235	306/322	219/231
	Gab-2413	−	/	/	08/04/13		170/174	237/237	179/179	302/302	231/235	306/322	219/231
OD-ID-07	Gab-2414	−	/	/	04/04/13	M	158/170	205/245	167/179	290/306	231/255	306/322	223/227
	Gab-2415	−	/	/	04/04/13		158/170	205/245	167/179	290/306	231/255	306/322	223/227
OD-ID-08	Gab-2420	−	/	/	02/04/17	F	162/166	205/225	167/187	298/302	235/243	322/322	219/227
	Gab-2421	−	/	/	02/04/17		162/166	205/225	167/187	298/302	235/243	322/322	219/227
OD-ID-09	Gab-2425	−	/	/	08/04/13	M	154/162	225/245	179/183	298/302	243/255	306/330	227/227
	Gab-2426	−	/	/	08/04/13		154/162	225/245	179/183	298/302	243/255	306/330	227/227
	Gab-2427	−	/	/	08/04/13		154/162	225/245	179/183	298/302	243/255	306/330	227/227
OD-ID-10	Gab-2428	+	−	−	08/04/13	M	154/166	225/237	175/179	298/302	251/255	306/318	219/227
OD-ID-11	Gab-2429	−	/	/	08/04/13	M	146/150	173/197	191/191	282/286	247/255	330/338	227/247
OD-ID-12	Gab-2430	−	/	/	08/04/13	F	146/146	185/225	179/ -	274/298	247/255	306/ -	219/219
	Gab-2431	−	/	/	08/04/13		146/146	185/225	179/179	274/298	247/ -	306/306	219/219
	Gab-2432	−	/	/	08/04/13		146/146	185/225	179/179	274/298	247/255	306/306	219/219
OD-ID-13	Gab-2433	−	/	/	08/04/13	F	166/174	225/229	175/183	274/302	235/259	314/318	231/231
	Gab-2434	−	/	/	08/04/13		166/174	225/229	175/183	274/302	235/259	314/318	231/231
OD-ID-14	Gab-2435	−	/	/	08/04/13	F	-/ -	225/229	175/183	302/302	215/ -	-/ -	231/231
OD-ID-15	Gab-2436	−	/	/	08/04/13	M	134/170	225/225	171/179	-/298	235/235	306/306	219/243
	Gab-2437	−	/	/	08/04/13		134/170	225/225	171/179	298/298	235/235	306/306	219/243
OD-ID-16	Gab-2438	−	/	/	08/04/13	F	146/150	185/221	167/179	298/314	235/255	306/310	219/219
	Gab-2439	−	/	/	08/04/13		146/150	185/221	167/179	298/314	235/255	306/310	219/219
	Gab-2440	+	−	−	08/04/13		146/150	185/221	167/179	298/314	235/255	306/310	219/219
	Gab-2441	−	/	/	11/04/13		146/150	185/221	167/179	298/314	235/255	306/310	219/219
OD-ID-17	Gab-2442	+	−	−	11/04/13	F	138/162	229/229	167/175	298/310	223/231	306/322	215/231

Table S2. *pol* and *env* genes primers.

Fragment	Primers [a]	SIZE (bp)
Env (gp41)/nef		
First round	GP40F1 (5′-TTAGGCAGGGATACTCACCTTTGTCGTT-3′)	
	GP41R1 (3′-TCTTAGGAGCAGCGGAAGCACTATGGG-5′)	
Second round	gp46F2 (5′-ACAATTATTGTCTGGTATAGTGCAACAGCA-3′)	
	gp48R2 (5′-TCCTACTATCATTATGAATATTTTTATATA-3′)	400
First round	Mak-gp41-F1 (5′-AGCCATAGAAGCGCAACAACA-3′)	
	Mak-gp41-R1 (3′-TCCAAAGGCTGTCCCATTGACT-5′)	
Second round	Mak-gp41-F2 (5′-AAGCTCGATTACTTGCTGTGGA-3′)	
	Mak-gp41-R2 (3′-TGTGCTGCTTCTAATAATCCA-5′)	200
First round	Mak-gp41-F1	
	GP41NEFR1 (3′-CCCWTCCAGTCCCCCCTTTCC-5′)	
Second round	Mak-gp41-F2	
	GP41NEFR2 (3′-TCCCCCCTTTTCTTTTAAAAA-5′)	900
First round	Mak-gp120-F1 (5′-ATCCAACAGGGGGAGASATGASAG-3′)	
	Mak-gp41-R1	
	Mak-gp120-F2 (5′- GCCTTTGGACTRGGAGCACTGTTC-3′)	
	Mak-gp41-R2	400
Pol		
First round	POLIS 4 (5′-CCAGCNCACAAAGGNATAGGAGG-3′)	
	POLOR (3′-ACBACYGCNCCTTCHCCTTTC-5′)	
Second round	POLIS2 (5′-TGGCARATRGAYTGYACNCAYNTRGAA-3′)	
	UNI2 (3′-CCCCTATTCCTCCCCTTCTTTTAAAA-5′)	400
First round	Mak-pol-F1 (5′-AGAAGCAGAAGTAATACCAGCAGA-3′)	
	Mak-pol-R1 (3′-TGAAGACTGCCATTTGTACTGC-5′)	
Second round	Mak-pol-F2 (5′-AGAAACTGGGCAGGAAACA-3′)	
	Mak-pol-R2 (3′-TGGATTCTACCACTCCTTGACT-5′)	200
First round	Mak-pol-F1	
Second round	Mak-pol-F2	
	Mak-pol-R4 (3′- CAGKAGMGTTGCTGGTCCTTTCC-5′)	400

[a] Y= C ou T; W= A ou T; R= A ou G; H= A ou C ou T; B= C ou G ou T; M= A ou C; S= G ou C; K= G ou T; V= G ou A ou C; D= G ou A ou T; N= A ou G ou C ou T, I= inosine.

Table S3. List of individuals with parental relations (LOD score at 95% confidence). In bold: SIV+ individuals. Lines in grey highlight pair of individuals both infected with SIV.

Offspring ID	Sex	Loci typed	Candidate ID	Sex	Loci typed	Pair loci compared	Pair loci mismatching	Pair LOD score 95% confidence
**MA-ID-003**	**F**	6	**MA-ID-077**	**F**	7	6	0	9.20631263573333E+0000
**MA-ID-011**	**F**	7	MA-ID-106	M	6	6	0	8.71732786431274E+0000
**MA-ID-016**	**M**	7	**MA-ID-066**	**M**	7	7	0	1.03258696694692E+0001
**MA-ID-019**	**M**	7	MA-ID-105	M	6	6	0	1.06164413824542E+0001
**MA-ID-024**	**M**	7	MA-ID-119	F	7	7	0	9.03188969324528E+0000
**MA-ID-025**	**F**	7	MA-ID-108	M	7	7	0	8.64547545182475E+0000
**MA-ID-031**	**M**	6	**MA-ID-066**	**M**	7	6	0	9.52953898624894E+0000
**MA-ID-042**	**M**	7	MA-ID-112	F	7	7	0	7.42363745044281E+0000
**MA-ID-045**	**M**	7	MA-ID-093	M	7	7	0	7.63167387777519E+0000
**MA-ID-059**	**M**	7	**MA-ID-063**	**F**	7	7	0	7.94735817350110E+0000
**MA-ID-061**	**F**	7	**MA-ID-191**	**M**	7	7	0	8.22965964966609E+0000
**MA-ID-062**	**M**	7	MA-ID-135	M	7	7	0	7.45696284268206E+0000
**MA-ID-063**	**F**	7	**MA-ID-059**	**M**	7	7	0	7.94735817350110E+0000
**MA-ID-066**	**M**	7	**MA-ID-016**	**M**	7	7	0	1.03258696694692E+0001
**MA-ID-067**	**M**	7	MA-ID-151	M	7	7	0	7.40396412882428E+0000
**MA-ID-077**	**F**	7	**MA-ID-003**	**F**	6	6	0	9.20631263573333E+0000
MA-ID-082	F	7	MA-ID-152	F	7	7	0	9.61822896769296E+0000
MA-ID-089	M	7	MA-ID-092	F	7	7	0	7.90355114867577E+0000
MA-ID-092	F	7	MA-ID-089	M	7	7	0	7.90355114867577E+0000
MA-ID-093	M	7	**MA-ID-045**	**M**	7	7	0	7.63167387777519E+0000
MA-ID-105	M	6	**MA-ID-019**	**M**	7	6	0	1.06164413824542E+0001
MA-ID-106	M	6	**MA-ID-011**	**F**	7	6	0	8.71732786431274E+0000
MA-ID-108	M	7	**MA-ID-025**	**F**	7	7	0	8.64547545182475E+0000
MA-ID-110	M	6	MA-ID-105	M	6	6	0	1.02931044631218E+0001
MA-ID-112	F	7	**MA-ID-042**	**M**	7	7	0	7.42363745044281E+0000
MA-ID-119	F	7	**MA-ID-024**	**M**	7	7	0	9.03188969324528E+0000

Table S3. *Cont.*

Offspring ID	Sex	Loci typed	Candidate ID	Sex	Loci typed	Pair loci compared	Pair loci mismatching	Pair LOD score 95% confidence
MA-ID-125	F	7	MA-ID-190	F	7	7	0	1.02453660493331E+0001
**MA-ID-126**	**M**	7	**MA-ID-191**	**M**	7	7	0	1.06373885013764E+0001
**MA-ID-129**	**M**	7	MA-ID-211	F	7	7	0	8.76633053818609E+0000
**MA-ID-132**	**M**	7	**MA-ID-210**	**F**	7	7	0	8.04771000599110E+0000
MA-ID-135	M	7	**MA-ID-062**	**M**	7	7	0	7.45696284268206E+0000
MA-ID-140	M	7	MA-ID-196	F	7	7	0	8.76273390364319E+0000
MA-ID-146	F	7	MA-ID-147	F	7	7	0	7.94002177333959E+0000
MA-ID-147	F	7	MA-ID-146	F	7	7	0	7.94002177333959E+0000
MA-ID-151	M	7	**MA-ID-067**	**M**	7	7	0	7.40396412882428E+0000
MA-ID-152	F	7	MA-ID-082	F	7	7	0	9.61822896769296E+0000
**MA-ID-164**	**F**	5	OD-ID-05	F	4	4	0	7.36133316452040E+0000
MA-ID-190	F	7	MA-ID-125	F	7	7	0	1.02453660493331E+0001
**MA-ID-191**	**M**	7	**MA-ID-126**	**M**	7	7	0	1.06373885013764E+0001
MA-ID-196	F	7	MA-ID-140	M	7	7	0	8.76273390364319E+0000
MA-ID-200	M	7	**MA-ID-202**	**F**	7	7	0	8.39781646625790E+0000
**MA-ID-202**	**F**	7	MA-ID-200	M	7	7	0	8.39781646625790E+0000
**MA-ID-210**	**F**	7	**MA-ID-132**	**M**	7	7	0	8.04771000599110E+0000
MA-ID-211	F	7	**MA-ID-129**	**M**	7	7	0	8.76633053818609E+0000
MA-ID-208	M	7	**MA-ID-129**	**M**	7	7	0	8.00648375493281E+0000
**ML-ID-01**	**F**	7	**ML-ID-04**	**F**	7	7	0	7.35898553068168E+0000
**ML-ID-03**	**F**	7	**ML-ID-06**	**F**	7	7	0	9.96649430023361E+0000
**ML-ID-04**	**F**	7	**ML-ID-08**	**F**	7	7	0	8.69085564946904E+0000
**ML-ID-06**	**F**	7	**ML-ID-03**	**F**	7	7	0	9.96649430023361E+0000
**ML-ID-08**	**F**	7	**ML-ID-04**	**F**	7	7	0	8.69085564946904E+0000
ML-ID-10	M	7	**ML-ID-12**	**M**	7	7	0	1.49649746398789E+0001
**ML-ID-12**	**M**	7	ML-ID-10	M	7	7	0	1.49649746398789E+0001

Table S3. *Cont.*

Offspring ID	Sex	Loci typed	Candidate ID	Sex	Loci typed	Pair loci compared	Pair loci mismatching	Pair LOD score 95% confidence
ML-ID-15	M	7	ML-ID-19	M	7	7	0	1.19097804392944E+0001
ML-ID-19	M	7	ML-ID-15	M	7	7	0	1.19097804392944E+0001
IY-ID-01	M	7	IY-ID-02	M	4	4	0	8.34143968584609E+0000
IY-ID-02	M	4	IY-ID-01	M	7	4	0	8.34143968584609E+0000
OD-ID-02	F	7	OD-ID-03	M	5	5	0	9.17690161329226E+0000
OD-ID-03	M	5	OD-ID-02	F	7	5	0	9.17690161329226E+0000
OD-ID-05	F	4	**MA-ID-164**		5	4	0	7.36133316452040E+0000
